# Electroactive biomaterials modulating ion channels in nervous system regeneration

**DOI:** 10.1093/rb/rbag168

**Published:** 2026-08-04

**Authors:** Zide Wang, Adilijiang Aihemaitiniyazi, Toshitatsu Nagayasu, Yi Lu, Hong Guo, Jianhang Zhang, Shenglian Yao, Guihuai Wang, Weitao Man

**Affiliations:** Department of Neurosurgery, Beiing Tsinghua Changgung Hospital, School of Clinical Medicine, Tsinghua Medicine, Tsinghua University, Beijing 102218, China; Department of Neurosurgery, Brigham and Women’s Hospital, Harvard Medical School, Boston, MA 02115, USA; Department of Neurosurgery, Beiing Tsinghua Changgung Hospital, School of Clinical Medicine, Tsinghua Medicine, Tsinghua University, Beijing 102218, China; State Key Laboratory of New Ceramic Materials, Key Laboratory of Advanced Materials of Ministry of Education, School of Materials Science and Engineering, Tsinghua University, Beijing 100084, China; Department of Neurosurgery, Brigham and Women’s Hospital, Harvard Medical School, Boston, MA 02115, USA; Department of Neurosurgery, Brigham and Women’s Hospital, Harvard Medical School, Boston, MA 02115, USA; Department of Neurosurgery, Beiing Tsinghua Changgung Hospital, School of Clinical Medicine, Tsinghua Medicine, Tsinghua University, Beijing 102218, China; School of Materials Science and Engineering, University of Science and Technology Beijing, Beijing 100083, China; Department of Neurosurgery, Beiing Tsinghua Changgung Hospital, School of Clinical Medicine, Tsinghua Medicine, Tsinghua University, Beijing 102218, China; Department of Neurosurgery, Beiing Tsinghua Changgung Hospital, School of Clinical Medicine, Tsinghua Medicine, Tsinghua University, Beijing 102218, China; State Key Laboratory of New Ceramic Materials, Key Laboratory of Advanced Materials of Ministry of Education, School of Materials Science and Engineering, Tsinghua University, Beijing 100084, China

**Keywords:** conductive materials, piezoelectric materials, spinal cord injury, traumatic brain injury, peripheral nerve injury, nerve regeneration

## Abstract

Traumatic injury to the central and peripheral nervous systems disrupts the electrophysiological microenvironment through persistent ionic dysregulation and maladaptive ion channel remodeling, creating conditions that are fundamentally hostile to endogenous repair. Conventional biomaterials, while offering structural support, remain electrically inert and unable to reconstitute the bioelectric signaling context essential for functional neural regeneration. Here, we review the emerging class of electroactive biomaterials, encompassing conductive and piezoelectric platforms that are engineered to actively interface with and therapeutically remodel this pathological milieu. We first systematically characterize the electrophysiological sequelae of neurotrauma, detailing how glutamate excitotoxicity, GABAergic polarity inversion and voltage-gated channel dysfunction collectively drive aberrant depolarized states in both central and peripheral injury contexts. We then analyze how electroactive scaffolds transduce or autonomously generate localized electrical cues to modulate ion channel kinetics and activate neurogenic cascades across brain, spinal cord and peripheral nerve injury models. Finally, we critically examine translational barriers—including interfacial impedance mismatch, asynchronous biodegradation and neuroimmune reactivity—that currently impede clinical deployment. This synthesis identifies key design imperatives for next-generation bioelectronic therapies capable of dynamically restoring electrophysiological homeostasis to promote meaningful neural recovery.

## Graphical abstract

**Graphical Abstract:**
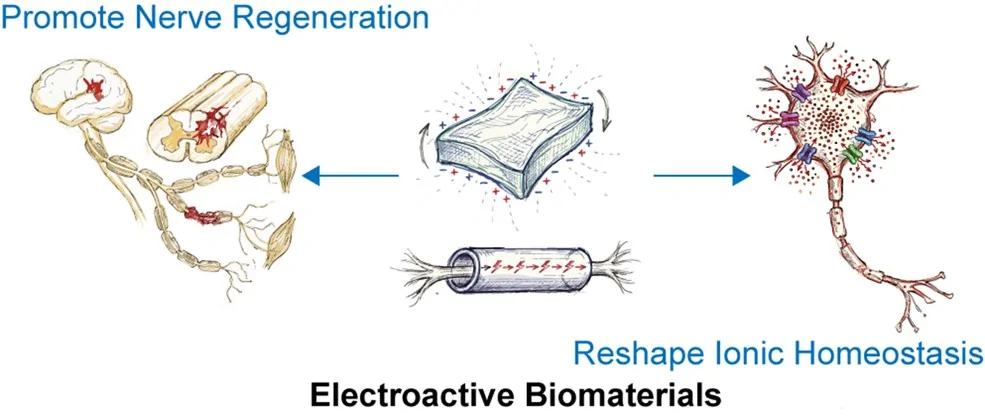

## Introduction

Neural function depends on the precise maintenance of transmembrane ionic gradients encoding information, sustaining action potential propagation and calibrating the excitation/inhibition (E/I) balance that governs network stability in the central and peripheral nervous systems (CNS and PNS) [1–5]. This electrochemical architecture functions as a highly dynamic, inherently unstable state, requiring continuous metabolic investment to coordinate voltage-gated ion channels, chloride cotransporters and synaptic machinery [6–9]. The peripheral nervous system extends this framework to somatic and autonomic targets, where the firing properties of sensory populations are similarly defined by voltage-gated channel repertoires [9, 10]. Traumatic insults to the nervous system including spinal cord injury (SCI), traumatic brain injury (TBI) and peripheral nerve injury (PNI), contribute to the irreversible collapse of the local electrophysiological microenvironment [11–14]. The secondary injury cascade that follows primary trauma is well described, but existing treatments inadequately target the persistent ionic dysregulation driving chronic pathology [15]. In the CNS, glutamate-induced Ca^2+^ overload predictably triggers excitotoxicity [16]. However, a more insidious and structurally disruptive mechanism involves the functional inversion of GABAergic signaling; the degradation of the K^+^-Cl^−^ cotransporter 2 (KCC2)/Na^+^-K^+^-2Cl^−^ cotransporter 1 (NKCC1) cotransporter axis forces neural networks into a pathologically depolarized state, catastrophically destabilizing the E/I balance [7, 17]. Conversely, PNS trauma initiates pathogenic remodeling of voltage-gated sodium and potassium channels, generating persistent conduction blocks and ectopic discharges [18, 19]. Collectively, these ion-channel derangements dictate a hostile microenvironment that actively antagonizes spontaneous axonal regeneration and functional recovery [15].

Conventional hydrogels and electrospun scaffolds provide topographical guidance and mechanical support, but they are electrically inert. They stabilize the structural defect without restoring the bioelectric context on which neural repair depends. This functional gap is the central problem that electroactive biomaterials are designed to solve. Electroactive biomaterials encompassing both conductive and piezoelectric platforms have emerged as dynamic modulators in neural regeneration [2]. Conductive biomaterials, including intrinsically conducting polymers and carbon-based nanomaterials, transmit applied or endogenous electrical signals across lesion gaps, directly modulating voltage-gated channel activity, intracellular Ca²^+^ dynamics and downstream neurogenic cascades [20–24]. Alternatively, piezoelectric materials transduce ambient mechanical deformation into local electrical polarization, providing wireless bioelectric stimulation without tethered electrodes or external power sources. By coupling to ultrasound or cyclic physiological strain, they can deliver spatially targeted cues to deep or structurally complex lesion sites [25–28]. These platforms couple topographical guidance with active signal transduction, modulating ion channel kinetics and engaging downstream neurogenic cascades. However, their clinical translation remains hampered by a superficial understanding of *in vivo* electro-ionic integration.

While recent reviews have extensively surveyed electroactive biomaterials for neural repair, they predominantly fragment the field by focusing on specific material classes or individual injury types [29–32]. Moving beyond a conventional catalogue of material innovations, this review integrates SCI, TBI and PNI to demonstrate how shared, injury-driven ionic dysregulation should directly dictate electroactive material design. To construct this framework, we begin by outlining the ion channel alterations that define the electrophysiological landscape of CNS and PNS trauma. Building on this pathological context, we evaluate conductive and piezoelectric platforms across brain, spinal cord and peripheral nerve repair applications, with a focus on mechanistic evidence, model limitations and reproducibility. Finally, we identify the translational bottlenecks, including interface instability, degradation mismatch and neuroimmune interactions, which must be resolved before these materials can successfully transition from bench to bedside.

## Electrophysiological and ionic alterations following central and peripheral nerve trauma

### Normal electrophysiology and ionic homeostasis in the central nervous system

The information-processing capacity of the CNS fundamentally depends on the precise regulation of neuronal excitability and the equilibrium of synaptic networks [33]. This functional integrity is rooted in the maintenance of electrochemical homeostasis, including the tightly controlled transmembrane gradients of calcium (Ca^2+^), chloride (Cl^−^), sodium (Na^+^) and potassium (K^+^) ions, which are essential for physiological neuronal signaling, action potential propagation and circuit stability [33–36]. At the core of CNS communication lies the interplay between two principal neurotransmitter systems: glutamatergic transmission, which mediates fast excitation, and gamma-aminobutyric acid (GABA)ergic transmission, which provides the primary mechanism of fast synaptic inhibition [8, 37, 38]. The precise tuning of these counteracting forces establishes the E/I balance that governs network activity, synchronizes oscillations and regulates behavioral outputs [8, 39, 40].

#### Glutamatergic excitation

Glutamate serves as the principal excitatory neurotransmitter in the mature mammalian CNS [37, 41]. Upon action potential arrival at the presynaptic terminal, vesicular glutamate is released into the synaptic cleft via Ca^2+^-dependent exocytosis, subsequently binding to ionotropic and metabotropic glutamate receptors [41]. Ionotropic glutamate receptors (iGluRs), ligand-gated ion channels mediating rapid synaptic transmission, are classified into N-methyl-D-aspartate (NMDA) [42], α-amino-3-hydroxy-5-methyl-4-isoxazolepropionic acid (AMPA) [43] and kainate (KA) [44] subtypes. AMPA and KA receptors predominantly mediate rapid postsynaptic depolarization through a net inward cationic current carried primarily by Na^+^ influx, whereas NMDA receptors exhibit distinctive biophysical properties, including high Ca^2+^ permeability and a voltage-dependent magnesium (Mg^2+^) block at resting membrane potentials [17, 45, 46]. Coincident postsynaptic depolarization, driven by AMPA receptor activation, relieves this Mg^2+^ block, permitting substantial Ca^2+^ influx [42]. This Ca^2+^ influx functions as a key second messenger, engaging downstream signaling cascades including calcium/calmodulin-dependent protein kinase II (CaMKII) and mitogen-activated protein kinase (MAPK) pathways [46]. Activated CaMKII directly phosphorylates AMPA receptors and promotes their lateral trafficking into the postsynaptic density, a molecular hallmark of long-term potentiation and memory consolidation [46].

#### GABAergic inhibition

Concurrently, GABA serves as the primary inhibitory neurotransmitter, providing a counterbalance to suppress hyperexcitability and refine neural plasticity [7, 8]. GABAergic signaling is transduced through two major receptor classes: the ionotropic GABAA receptors (GABAARs), including the pharmacologically distinct GABAA-ρ subpopulation, and the metabotropic GABAB receptors (GABABRs) [47]. GABAARs are ligand-gated heteropentameric Cl^−^ channels that mediate rapid phasic and tonic inhibition [48, 49]. In the mature, uninjured nervous system, the prevailing transmembrane electrochemical gradient for Cl^−^ drives a net Cl^−^ influx upon GABAAR opening, leading to postsynaptic membrane hyperpolarization and a diminished probability of action potential generation [49]. By contrast, GABABRs are G-protein-coupled receptors that elicit a slower, sustained inhibitory tone by repressing adenylyl cyclase activity, inhibiting voltage-gated Ca^2+^ channels and simultaneously activating G-protein-coupled inwardly rectifying K^+^ channels to stabilize the resting membrane potential [50].

#### Chloride homeostasis

The efficacy and polarity of GABAAR-mediated inhibition are contingent upon maintenance of the neuronal intracellular chloride gradient, which is governed by two primary cation–chloride cotransporters: the NKCC1, which drives active Cl^−^ accumulation, and the neuron-specific KCC2, which extrudes Cl^−^ against its electrochemical gradient [38, 51]. In the mature CNS, robust KCC2 expression coupled with low NKCC1 activity maintains a low intracellular chloride concentration ([Cl^−^]ᵢ), guaranteeing hyperpolarizing GABA responses [52]. The balance of glutamatergic and GABAergic networks, supplemented by neuromodulatory inputs of monoamines (e.g. dopamine and serotonin) and acetylcholine, alongside glial homeostatic support such as astrocytic inwardly rectifying K^+^ Kir4.1 channels that mediate spatial potassium buffering—is vital for sustaining rhythmic central pattern generator (CPG) outputs and preventing circuit hyperexcitability [6, 36].

### Electrophysiology and ionic imbalance following central nervous system injury

Traumatic insults to the CNS, encompassing SCI and TBI, remain leading global causes of permanent neurological disability [16, 53, 54]. Although the initial mechanical impact causes immediate, irreversible structural disruption and necrotic cell death, it is the secondary injury cascade that drives progressive tissue loss, neuronal apoptosis and circuit disruption [15]. A central feature of this secondary pathophysiology is the collapse of transmembrane ionic gradients and the consequent pathological E/I imbalance (Figure 1) [15, 39].

**Figure 1:**
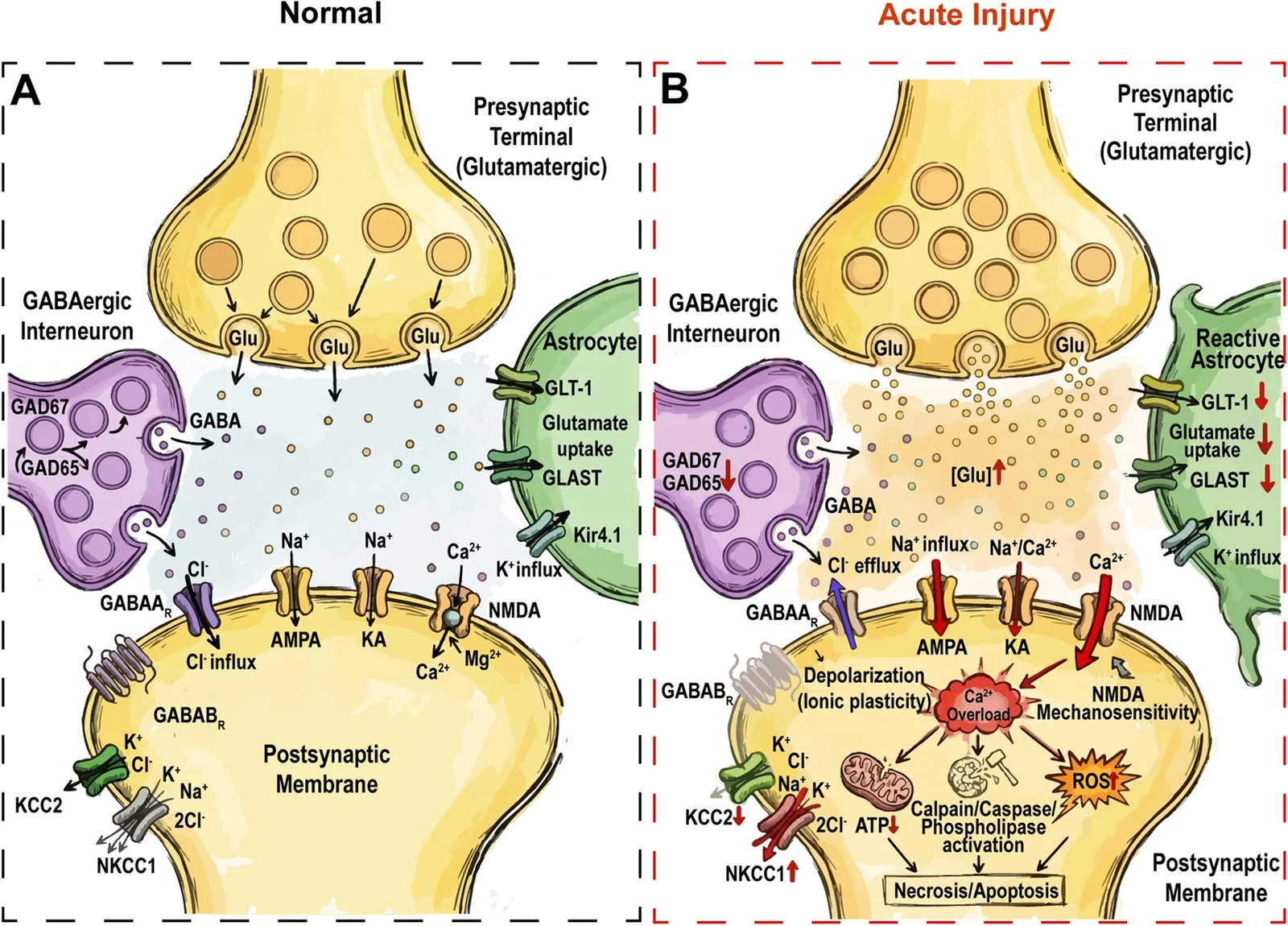
Disruption of ionic and neurotransmitter homeostasis following CNS injury. (**A**) Normal CNS ionic homeostasis. In the mature, uninjured CNS, glutamatergic excitation and GABAergic inhibition are precisely balanced to maintain circuit stability. Presynaptic glutamate release activates three classes of postsynaptic ionotropic glutamate receptors (iGluRs): AMPA and kainate (KA) receptors, which mediate fast depolarization via net Na^+^ influx, and NMDA receptors, which exhibit high Ca^2+^ permeability following voltage-dependent Mg^2+^ block relief upon coincident AMPA-driven depolarization. Ca^2+^-dependent CaMKII activation subsequently drives AMPA receptor phosphorylation and lateral trafficking into the postsynaptic density, constituting the molecular basis of LTP. GABAergic inhibition is transduced through two receptor classes: ionotropic GABAARs, which drive Cl^−^ influx and membrane hyperpolarization, and metabotropic GABABRs, which elicit a slower, sustained inhibitory tone by suppressing adenylyl cyclase activity, inhibiting voltage-gated Ca^2+^ channels and activating GIRK channels to stabilize the resting membrane potential. The inhibitory polarity of GABAAR-mediated signaling is maintained by robust KCC2-mediated Cl^−^ extrusion coupled with low NKCC1 activity, collectively sustaining a low [Cl^−^]ᵢ. Astrocytic GLT-1/GLAST transporters and Kir4.1-mediated K^+^ spatial buffering provide essential glial homeostatic support. (**B**) Pathological ionic changes following CNS acute injury. Traumatic insult initiates a secondary injury cascade driven by the collapse of ionic homeostasis. (**i**) Glutamate excitotoxicity: massive glutamate efflux from mechanically ruptured neurons, compounded by energy failure-driven downregulation of astrocytic GLT-1 and GLAST, drives sustained overactivation of all three iGluR subtypes—AMPA, KA and NMDA receptors—triggering pathological Ca^2+^ overload; the intrinsic mechanosensitivity of NMDA receptors further lowers their activation threshold under mechanical shear forces, amplifying Ca^2+^ influx; this intracellular Ca^2+^ accumulation is further amplified by the recruitment of voltage-dependent N-type (CaV2.2) and P/Q-type (CaV2.1) Ca^2+^ channels, collectively activating calpains, caspases and phospholipases, collapsing mitochondrial membrane potential, impairing ATP synthesis and generating ROS, ultimately culminating in necrotic and apoptotic neuronal death. (**ii**) GABAergic dysfunction and ionic plasticity: transcriptional downregulation of KCC2 coupled with concurrent NKCC1 upregulation elevates [Cl^−^]ᵢ and shifts E_Cl_ to depolarizing values, functionally converting GABAAR-mediated signaling from hyperpolarizing inhibition to depolarizing excitation; chronic inhibitory capacity is further undermined by the downregulation of the GABA-synthesizing enzymes GAD65 and GAD67, despite a transient acute rise in extracellular GABA levels in the immediate post-injury period.

#### Glutamate excitotoxicity

The acute phase of both SCI and TBI is defined by a pathological accumulation of extracellular glutamate within the extracellular space [55, 56]. Under physiological conditions, extracellular glutamate concentrations are maintained at approximately 0.6 µmol/L, but following trauma they can increase nearly tenfold to around 10 µmol/L as a result of intracellular glutamate release from damaged neurons [57]. This increase arises from the mechanical rupture of cellular membranes and the downregulation or functional impairment of astrocytic excitatory amino acid transporters (EAATs, particularly GLT-1/GLAST) under conditions of energy failure [57–59]. Following TBI, both astrocytic glutamate transporters, GLT-1 and GLAST, undergo transient downregulation in the ipsilateral cortex [58]. In contrast, following SCI, GLT-1 expression at the injury epicenter remains significantly reduced for up to 56 days, whereas GLAST exhibits only a mild and transient decrease, with levels largely returning to baseline thereafter [59]. Chronic overstimulation of postsynaptic iGluRs by excess extracellular glutamate results in excitotoxic neuronal death, further exacerbated by the intrinsic mechanosensitivity of NMDA receptors, which lowers their activation threshold under mechanical shear forces [16]. Sustained activation of NMDA, AMPA and KA channels triggers a large influx of Ca^2+^ into the neuronal somatodendritic domain [60]. This calcium overload further depolarizes the neuronal membrane, thereby recruiting voltage-dependent calcium channels, such as N-type and P/Q-type channels, which amplify intracellular Ca^2+^ accumulation [61]. This intracellular Ca^2+^ overload activates a cascade of calcium-dependent enzymes (including calpains, caspases and phospholipases), disrupts mitochondrial membrane potential, compromises ATP synthesis, drives reactive oxygen species (ROS) generation, and ultimately culminates in necrotic and apoptotic cell death [62–64]. Neurons attempt to buffer excitotoxic stress by modifying receptor subunit composition, such as internalizing NMDA receptors or incorporating calcium-impermeable GluA2 subunits into AMPA receptors [63, 65]. Though these adaptive shifts successfully slow calcium influx and resist acute excitotoxicity, they are ultimately insufficient. The initial Ca^2+^ overload irreversibly disrupts cellular bioenergetics before remodeling completes. Consequently, despite these homeostatic responses, the execution of apoptotic pathways and subsequent neuronal death remain unavoidable.

#### GABAergic dysfunction

Concurrent with the onset of hyperexcitation, the inhibitory machinery undergoes a functional conversion, a phenomenon termed 'ionic plasticity’ that is fundamentally driven by the disruption of chloride homeostasis [39]. Neurotrauma induces a rapid, transcriptional and post-translational downregulation of KCC2 expression and membrane stability, coupled with concurrent upregulation and activation of NKCC1 in surviving perilesional neurons [66–70]. The synergistic effect of impaired KCC2-mediated Cl^−^ extrusion and enhanced NKCC1-mediated Cl^−^ influx causes a pathological accumulation of [Cl^−^]ᵢ, shifting the chloride equilibrium potential (E_Cl_) to a more positive value than the resting membrane potential [71, 72]. Consequently, GABA binding to GABAARs results in net Cl^−^ efflux rather than influx, functionally converting GABA from a hyperpolarizing inhibitor into a depolarizing signal [39].

Superimposed on this functional conversion is a complex, time-dependent disruption of ambient GABA availability that varies significantly by injury type and severity. In the acute phase of SCI, extracellular GABA levels exhibit a transient increase within 30–60 min post-injury, before rapidly returning to baseline or declining below baseline [73–75]. Conversely, TBI precipitates distinct temporal profiles of GABA metabolic dysregulation: mild TBI induces a temporary elevation of GABA that resolves within 24 h, whereas severe TBI triggers a prolonged, sustained elevation that persists for up to 120 h [76]. Despite these transient acute surges, the long-term capacity for GABA synthesis is severely compromised; both SCI and TBI lead to substantial downregulation of the key synthesizing enzymes, GAD65 and GAD67 [77–79]. This synthetic deficit is tied to the disruption of the glutamate–glutamine cycle. Under physiological conditions, GABA is synthesized via the conversion of glutamine into glutamate by glutaminase, which is subsequently decarboxylated into GABA by GAD65 and GAD67 [7]. However, a massive post-traumatic accumulation of extracellular glutamate, coupled with impaired astrocytic glutamate reuptake, depletes GABAergic terminals of the glutamine substrate needed for synthesis [39]. This astrocytic clearance failure effectively cuts off the upstream supply of GABA while simultaneously weakening the network’s overall GABA reuptake capacity. Following SCI, reactive astrocytes significantly upregulate monoamine oxidase B (MAOB), leading to elevated GABA synthesis via the putrescine oxidation pathway [80]. This astrocyte-derived GABA is tonically released into the extrasynaptic space, functioning as a suppressor of local BDNF/TrkB signaling and attenuating endogenous regenerative programs [80]. Counteracting this suppression through exogenous BDNF administration can synergistically upregulate KCC2 and specific GABAAR subunits, restoring the neuronal E/I balance and chloride homeostasis, thereby promoting neuronal survival and the functional reestablishment of disrupted spinal circuits [81]. From a therapeutic perspective, effective management of SCI and TBI demands careful consideration of GABAergic plasticity. Pharmacological strategies targeting neurotransmitter levels alone are insufficient; restoration of KCC2- and NKCC1-mediated chloride homeostasis represents a fundamental requirement for re-establishing functional inhibitory signaling. Equally important is the contribution of glial cells, whose roles in this process extend well beyond passive support.

### Normal electrophysiology and ionic homeostasis in the peripheral nervous system

The PNS serves as a critical bidirectional conduit, relaying sensorimotor and autonomic signals between the CNS and somatic or visceral targets [10, 82, 83]. Anatomically, peripheral nerves exhibit a hierarchical organization: individual nerve fibers are embedded within the endoneurium, bundled into discrete fascicles by the perineurium and collectively invested by an outer protective connective tissue sheath, the epineurium [84]. This structural organization maintains the unique microenvironmental ionic composition indispensable for high-fidelity action potential (AP) propagation.

The operational units of the PNS comprise functionally heterogeneous neuronal populations. Efferent motor and autonomic fibers utilize acetylcholine or norepinephrine to drive peripheral execution, whereas afferent sensory fibers transduce mechanical, thermal and chemical stimuli from specialized peripheral receptors into electrical signals [10]. The somata of these primary somatosensory afferents reside within the dorsal root ganglia (DRG), anatomically situated within the protective intervertebral foramina [85]. DRG neurons exhibit a classic pseudounipolar morphology, giving rise to a single stem axon that bifurcates into a peripheral branch innervating target organs and a central branch projecting into the spinal dorsal horn.

Based on somatic cross-sectional diameter and axonal myelination status, these sensory neurons are classified into distinct functional populations that dictate AP conduction velocity and fidelity [85]. Large- and medium-diameter neurons primarily mediate proprioception and mechanoreception, respectively. Their axons are ensheathed by myelin produced by Schwann cells (SCs), which restrict active transmembrane ionic fluxes to the unmyelinated nodes of Ranvier. This spatial configuration facilitates salutatory conduction, enabling rapid AP propagation at velocities ranging from 30 to 120 m/s (e.g. Aα and Aβ primary afferents). By contrast, small-diameter DRG neurons (mediating nociception and thermoreception) predominantly give rise to slowly conducting, unmyelinated C fibers, exhibiting conduction velocities of approximately 0.5–2 m/s [10, 86]. Ultimately, the baseline excitability and distinct firing patterns of these diverse sensory populations are governed by a repertoire of voltage-gated ion channels.

#### Voltage-gated sodium (NaV) channels

Voltage-gated sodium (NaV) channels are determinants of AP threshold and initiation [87]. Structurally, they exist as heteromeric complexes comprising a pore-forming α subunit encoded by the SCN gene family (NaV1.1–NaV1.9), and ancillary β subunits that modulate gating kinetics and plasma membrane trafficking [9, 18, 88]. Adult DRG neurons predominantly express NaV1.7 together with NaV1.1, NaV1.6, NaV1.8 and NaV1.9 [89–92], whereas NaV1.2, NaV1.3 and NaV1.5 are primarily associated with embryonic DRG development and are progressively downregulated after birth [93, 94]. Under physiological conditions, mature NaV channels are concentrated at high densities at the axon initial segment and the nodes of Ranvier, ensuring rapid Na^+^ influx upon threshold depolarization and transmission of sensory and nociceptive signals to the spinal cord [87, 95].

#### Voltage-gated potassium (KV) channels

Voltage-gated potassium (KV) channels are critical for driving membrane repolarization, regulating refractory periods and establishing the resting membrane potential [19]. The neuronal KV channel superfamily is diverse, encompassing the Shaker-related KV1 subfamily, the Shab-related KV2 subfamily, the Shaw/Shal-related KV3 and KV4 subfamilies, the KCNQ (KV7) family and the EAG-related potassium channel family (KV10–KV12), although the functions of EAG-related channels in peripheral sensory neurons remain poorly characterized [96–99]. Within peripheral sensory networks, KV channels operate as hyperpolarizing regulators of inward depolarizing NaV and CaV currents [19]. Low-threshold KV1.1 and KV1.2 subunits regulate AP firing thresholds and frequency accommodation, whereas the delayed-rectifier KV2.1 and KV2.2 channels govern the kinetics of the AP repolarization phase [19]. Furthermore, the slow, non-inactivating M-current mediated by heteromeric KV7.2/KV7.3 (KCNQ2/3) complexes stabilizes the neuronal resting membrane potential below threshold, suppressing repetitive firing [100, 101].

#### Voltage-gated calcium (CaV) channels

Voltage-gated calcium (CaV) channels function as transducers, coupling membrane depolarization to intracellular Ca^2+^-dependent second messenger signaling and downstream biochemical cascades [102, 103]. Based on activation voltage thresholds, they are classified into low-voltage-activated (LVA; CaV3.1–CaV3.3, T-type) and high-voltage-activated (HVA; CaV1, L-type; CaV2.1, P/Q-type; CaV2.2, N-type and CaV2.3, R-type) channels [103–105]. HVA channels associate with regulatory accessory CaVβ and CaVα₂δ subunits that govern surface expression density and biophysical gating [104]. CaV2.2 channels are highly enriched at the presynaptic terminals of primary afferent central projections within the spinal superficial dorsal horn, where their opening triggers vesicular exocytosis of pro-nociceptive neurotransmitters, including glutamate and substance P [106, 107]. LVA T-type channels, particularly CaV3.2, activate near resting membrane potentials, driving subthreshold oscillations and burst firing that modulate the excitability of primary sensory somata [103]. Together, this integrated ion channel network ensures precise, adaptive signal processing across the intact PNS.

### Electrophysiology and ionic imbalance following peripheral nervous system injury

Traumatic PNI frequently results in long-term sensorimotor deficits and persistent neuropathic pain [86, 108]. Clinical data reveal a high co-occurrence of PNI and central neurotrauma: up to 60% of patients with mechanical PNI exhibit concomitant TBI, while approximately one-third of acute TBI patients sustain co-existing peripheral nerve lesions [109–111]. Following peripheral axotomy, a complex cascade of cellular and molecular events is initiated at the axonal lesion site, within the DRG microenvironment, and in central spinal circuits, resulting in remodeling of ion channel expression and function. Although these adaptive responses partially support axonal regeneration, they also contribute to neuronal hyperexcitability and neurodegeneration (Figure 2). In severe cases, axotomized motor neurons in the ventral horn and primary sensory DRG neurons undergo retrograde degeneration and apoptosis driven by oxidative stress, intracellular calcium overload and activation of pro-apoptotic signaling pathways, including Caspase-3, Caspase-8, TRAIL receptors and Fas-mediated death signaling [112].

**Figure 2:**
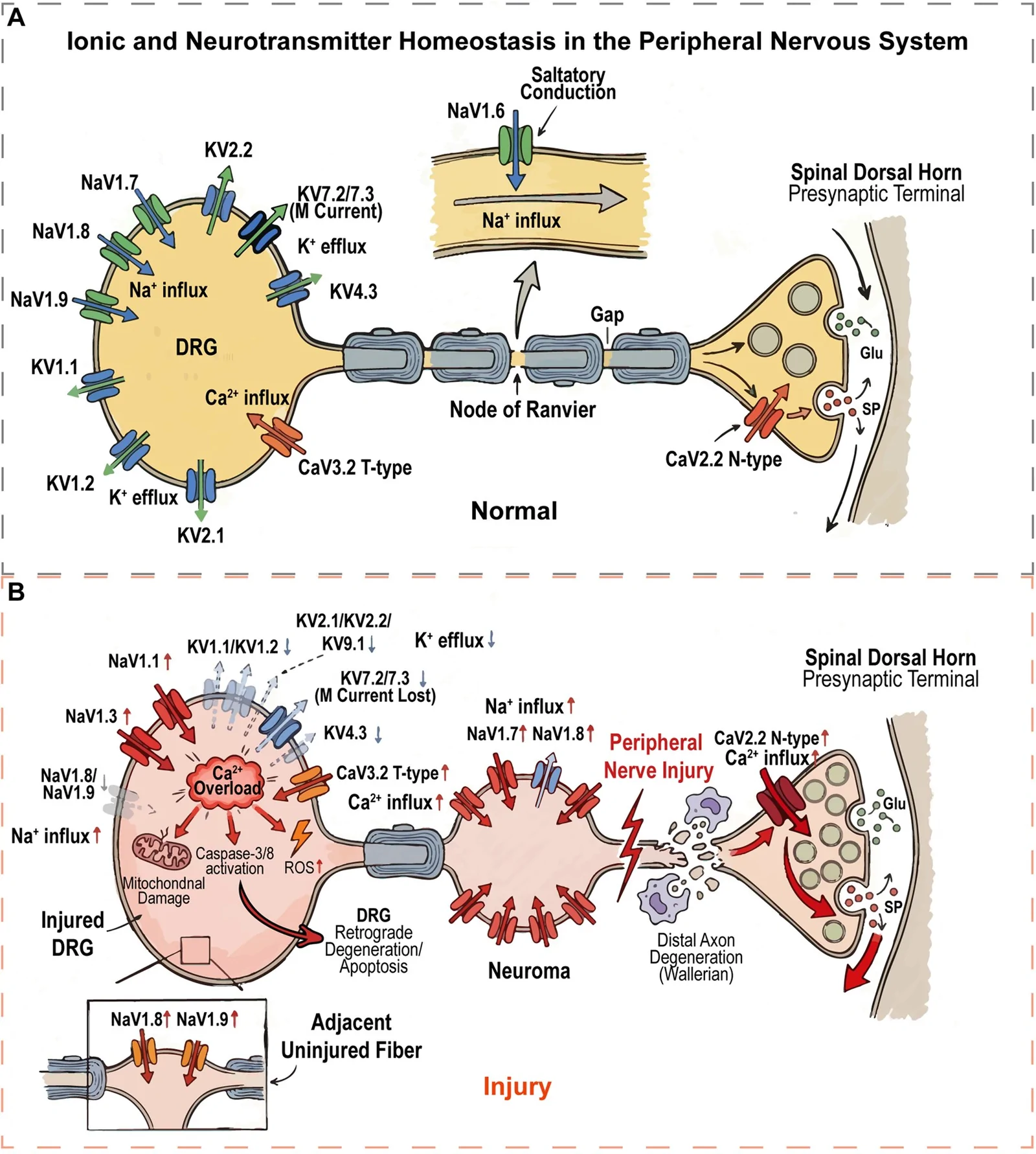
Peripheral nerve injury induces ion channel remodeling and neuronal hyperexcitability. (**A**) Normal PNS ion channel organization. In the intact peripheral nervous system, action potential initiation and propagation along primary sensory afferents depend on the precise spatial distribution of voltage-gated ion channels. NaV1.7, NaV1.8 and NaV1.9 are concentrated at the axon initial segment and nodes of Ranvier of DRG neurons, enabling threshold depolarization. KV1.1/1.2 and KV7.2/7.3 (M-current) stabilize the resting membrane potential and suppress repetitive firing. Presynaptic CaV2.2 (N-type) channels at central afferent terminals drive vesicular glutamate and substance P release into the spinal dorsal horn, while CaV3.2 (T-type) channels modulate subthreshold oscillations. Salutatory conduction along myelinated Aα/Aβ fibers is maintained by Schwann cell myelin sheaths confining ionic fluxes to nodes of Ranvier. (**B**) Pathological ion channel remodeling following peripheral nerve injury (PNI). Axotomy triggers a spatially distributed remodeling program across the DRG soma, the injury site/neuroma and adjacent uninjured fibers: (**i**) rapid re-expression of embryonic NaV1.3 and upregulation of NaV1.1 in injured DRG neurons promote ectopic discharge and mechanical allodynia, while NaV1.8/NaV1.9 are downregulated in axotomized segments but redistributed and upregulated in adjacent intact fibers; NaV1.7 and NaV1.8 accumulate at neuroma stumps, lowering local activation thresholds; (**ii**) KV channel loss, including epigenetic silencing of KV1.2, suppression of KV2.1/KV9.1, repression of KV7.2/7.3 M-current and downregulation of KV4.3, eliminates intrinsic hyperpolarizing control and facilitates high-frequency firing; (**iii**) upregulation of CaV2.2 at DRG somata and spinal dorsal horn terminals amplifies central sensitization via enhanced glutamate/substance P exocytosis, and Cdk5-dependent phosphorylation stabilizes surface CaV3.2, promoting burst firing. Upward arrows (↑) indicate upregulation or increase; downward arrows (↓) indicate downregulation or decrease. DRG, dorsal root ganglion; NaV, voltage-gated sodium channel; KV, voltage-gated potassium channel; CaV, voltage-gated calcium channel; SP, substance P; PNI, peripheral nerve injury.

#### Voltage-gated sodium (NaV) channels re-expression and redistribution

A hallmark of post-traumatic neuroplasticity is the extensive remodeling of NaV channel expression. Although adult DRG neurons normally exhibit minimal expression of the embryonic isoform NaV1.3, peripheral axotomy induces its rapid and robust re-expression through transcriptional upregulation [113–115]. This re-expression is strongly implicated in driving axonal hyperexcitability and ectopic discharges that underlie neuropathic mechanical allodynia [116]. Nevertheless, NaV1.3 null mutant mice continue to develop post-injury mechanical hypersensitivity, pointing to significant network redundancy and compensatory adaptations among remaining isoforms [117]. Concurrently, NaV1.1 expression undergoes upregulation within the injured DRG, further amplifying early aberrant electrical activity [118].

Conversely, the nociceptive isoforms NaV1.8 and NaV1.9 are markedly downregulated within axotomized segments. Adjacent uninjured fibers exhibit upregulation and redistribution of these same channels, generating a source of ectopic activity that amplifies pain transmission [119, 120]. Furthermore, NaV1.7 and NaV1.8 proteins accumulate at high densities within the proximal stumps of neuromas, lowering the local activation threshold and promoting spontaneous firing [119]. This cross-talk between injured and intact afferents, wherein localized variations in NaV1.8 kinetics modulate the activation threshold of adjacent NaV1.7-expressing fibers, constitutes a primary mechanism driving global mechanical hypersensitivity [121]. Focal neuroma accumulation is further accelerated by anterograde axonal transport and localized translation of NaV1.8 mRNA directly within peripheral axonal domains [122].

#### Voltage-gated potassium (KV) channels dysfunction

PNI consistently causes a profound reduction in the functional expression of stabilizing KV channels, removing key inhibitory regulation. KV1.2 expression in the DRG is silenced via epigenetic remodeling, including active DNA hypermethylation of the KCNA2 gene promoter driven by reduced TET1 demethylase activity, alongside microRNA miR-137-mediated post-transcriptional silencing [123, 124]. Similarly, the delayed-rectifier KV2.1 channel undergoes functional suppression post-injury, facilitating a transition toward high-frequency firing in response to sustained subthreshold inputs [125, 126]. The modulatory KV9.1 subunit, which governs KV2.1 inactivation kinetics, is likewise downregulated after PNI; experimental silencing of KV9.1 alone is sufficient to recapitulate neuropathic mechanical hypersensitivity [125, 127]. Furthermore, the M-current (KV7.2/KV7.3) is severely repressed following chronic constriction injury through a mechanism mediated by the upregulated repressor element-1 silencing transcription factor, which binds to neuron-restrictive silencer element domains at KCNQ loci to suppress transcription, thereby promoting hyperexcitability [128–131]. This is further compounded by the concurrent downregulation of transient low-threshold KV4.3 channels, resulting in a near-complete loss of intrinsic hyperpolarizing control [19].

#### Voltage-gated calcium (CaV) channels upregulation

Neurotrauma additionally induces substantial upregulation of HVA CaV2.2 channels. In rodent models of partial sciatic nerve ligation and chronic constriction injury, marked increases in CaV2.2α_1_ pore-forming protein density and expanded N-type current amplitudes are observed within primary DRG somata and their central synaptic projections in the spinal dorsal horn [132–134]. This enrichment facilitates primary afferent glutamate and substance P exocytosis, thereby driving central sensitization processes. Concurrently, LVA CaV3.2 T-type channels exhibit enhanced plasma membrane trafficking and stabilization, mediated by cyclin-dependent kinase 5 (Cdk5)-dependent phosphorylation, without alteration of total cellular protein expression [135, 136]. This expanded surface localization amplifies subthreshold calcium currents and promotes rhythmic burst firing. Antisense oligonucleotide or genetic silencing of CaV3.2 expression in primary sensory afferents successfully reverses neuropathic hyperalgesia, confirming its status as a validated therapeutic target [137, 138].

In summary, the pathophysiology of both CNS and PNS trauma is intrinsically linked to the failure of the electrophysiological microenvironment. Whether manifested as glutamate excitotoxicity and GABAergic dysfunction in the spinal cord, or ion channel remodeling and ectopic pacemaking in peripheral nerves, these ionic imbalances create a microenvironment that derails endogenous repair. Traditional pharmacological and biological interventions frequently fall short due to poor temporal-spatial precision and an inability to deliver the direct biophysical cues required to re-establish resting membrane potentials. Consequently, restoring electrical homeostasis has emerged as a prerequisite for functional neural regeneration. This gap paves the way for the application of electroactive biomaterials. By virtue of their tunable conductivity and dynamic tissue-interfacing capabilities, these advanced materials not only provide structural guidance for axonal extension but also actively modulate ion fluxes, buffer the injured microenvironment and deliver localized electrical stimulation (ES), ultimately bridging the gap between structural repair and electrophysiological recovery.

## Electroactive biomaterials in neural regeneration

Restoring bioelectric homeostasis is essential for neural regeneration [20, 22, 24]. Consequently, electroactive biomaterials have emerged as critical platforms to transmit, modulate, or autonomously generate electrical cues at the neural interface [24, 25, 27]. Based on their primary biophysical mechanisms, these materials are fundamentally classified into two complementary paradigms: conductive and piezoelectric biomaterials (Figure 3) [26, 139–141].

**Figure 3:**
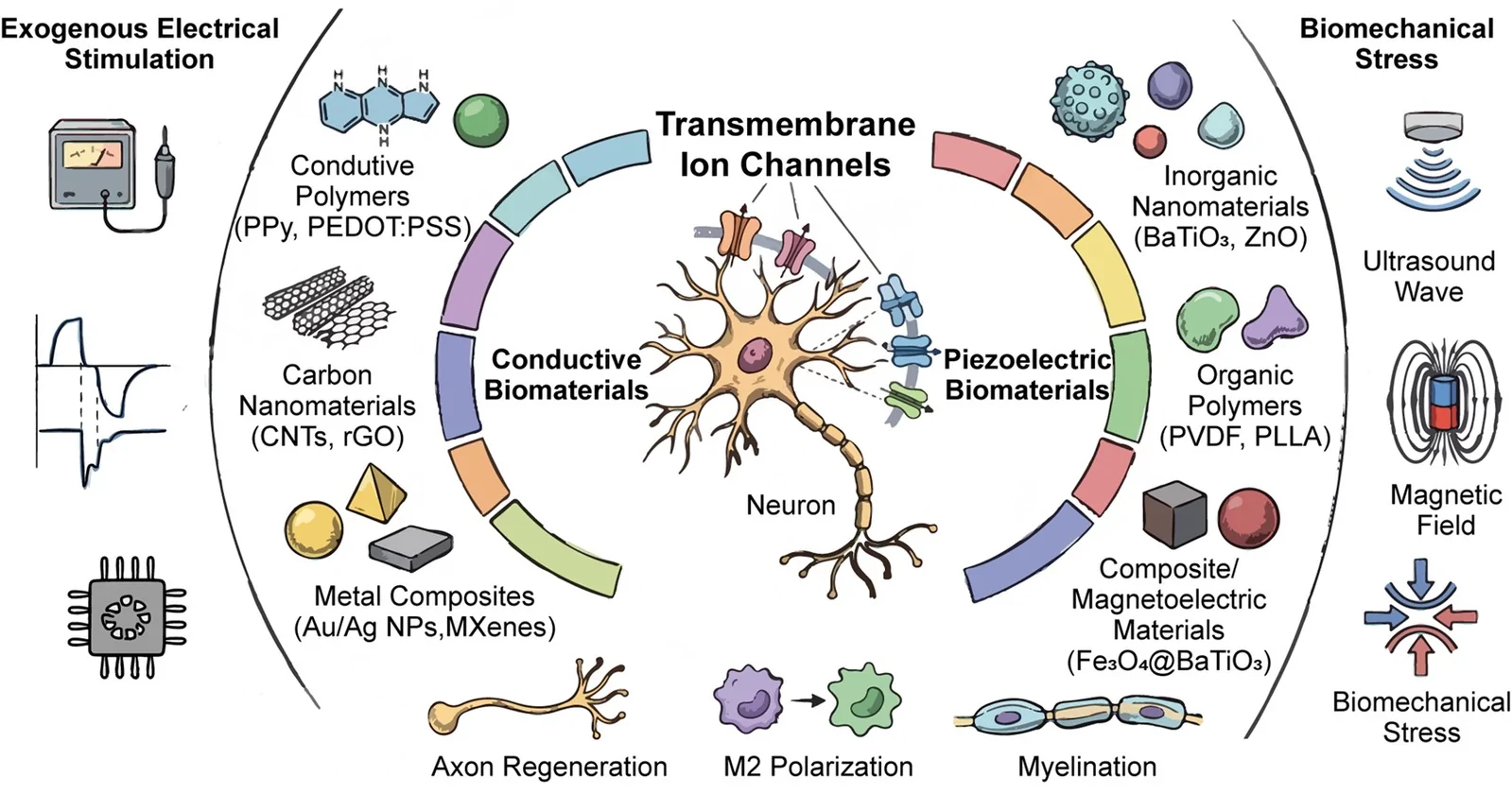
Schematic illustration of electroactive biomaterials for neural regeneration. Electroactive biomaterials restore bioelectric homeostasis at the neural interface by modulating transmembrane ion channels and activating intracellular regenerative signaling. Conductive biomaterials, including conductive polymers (PPy, PEDOT: PSS), carbon nanomaterials (CNTs, rGO) and metal-based composites (Au/Ag NPs, MXenes), transmit exogenous electrical stimulation from an external power source and electrode across non-conductive lesion gaps, delivering localized electrical cues to the neuron. Piezoelectric biomaterials, including inorganic nanomaterials (BaTiO_3_, ZnO), organic polymers (PVDF, PLLA) and magnetoelectric composites (Fe_3_O_4_@BaTiO_3_), convert physical triggers such as ultrasound, alternating magnetic fields and mechanical compression or tension into transient surface potentials that stimulate the neuron directly. These electroactive interfaces drive sequential histological outcomes, including directed neurite outgrowth for axon regeneration, macrophage M2 polarization for microenvironmental remodeling and targeted myelination for structural maturation.

### Conductive biomaterials

Conductive biomaterials transmit electrical cues to recapitulate the native neural electrophysiological microenvironment [22, 24]. Because neural cells are inherently electroresponsive, these materials directly modulate critical cellular processes, including proliferation, differentiation and directed neurite outgrowth [21]. Therapeutically, they act as active bridges across non-conductive lesion gaps, propagating endogenous action potentials and profoundly synergizing with exogenous ES [22]. These platforms are principally classified into three families: intrinsically conductive polymers (CPs), carbon nanomaterials and metallic composites [22, 24].

#### Categories and response mechanisms of conductive biomaterials

CPs feature a conjugated π-electron backbone enabling electron delocalization [24]. Pristine CPs range from insulating to semiconducting; achieving therapeutically relevant conductivities (up to 10³ S/cm) requires oxidative p-doping or reductive n-doping to generate mobile charge carriers [24, 142]. Clinically relevant CPs include polypyrrole (PPy) for its high conductivity, polyaniline (PANI) for tunable redox states and poly(3,4-ethylenedioxythiophene) polystyrene sulfonate (PEDOT: PSS) for superior aqueous processability [22, 23]. However, the clinical translation of pristine CPs remains restricted by their mechanical brittleness, limited machinability and dopant-induced cytotoxicity [24, 143].

Carbon nanomaterials, notably carbon nanotubes (CNTs) and graphene derivatives, offer outstanding electrical properties and high surface-area-to-volume ratios [22]. Graphene, a 2D sheet of sp²-hybridized carbon, exhibits superior in-plane conductivity. Its oxidized derivative, graphene oxide, facilitates aqueous dispersion at the expense of conductivity, which is partially restored via reduction to yield reduced graphene oxide (rGO) [144]. CNTs (single- or multi-walled) provide extreme aspect ratios and conductivities up to 10^5^ S/cm [145]. The primary translational bottleneck for carbon nanomaterials is their van der Waals-driven aggregation in physiological fluids, triggering chronic neuroinflammation [146]. Covalent or non-covalent surface functionalization with hydrophilic polymers is thus mandatory to confer colloidal stability and biocompatibility [147].

Metallic composites utilize gold (Au) or silver (Ag) nanoparticles as conductive fillers within insulating matrices. Unlike intrinsic continuous networks, these composites rely on percolation theory: bulk conductivity emerges only when the filler volume fraction exceeds the percolation threshold to establish interconnected pathways. High-aspect-ratio geometries (nanorods, nanowires) achieve this threshold at markedly lower concentrations, minimizing required filler loading [22]. Despite their synthetic versatility, long-term neurotoxicity remains a concern, particularly DNA intercalation by ultra-small Au nanoparticles (<1.4 nm) and the pro-inflammatory profile of Ag nanoparticles [148].

Mechanistically, conductive substrates surpass non-conductive controls by promoting focal adhesion and directed neurite extension—processes profoundly amplified by exogenous ES to accelerate neural differentiation and network synchronization [22]. Rather than passive conduits, these platforms act as active bioelectric modulators at the cellular interface. They trigger integrin-dependent mechanotransduction (e.g. the β1-integrin/FAK/ERK axis) and depolarize membranes to augment Ca^2+^ influx, thereby engaging downstream PKC/ERK pathways to drive the cytoskeletal and transcriptional remodeling essential for neural maturation [149]. PEDOT: PSS and carbon-based composites typically deliver charge densities of tens to hundreds of μC/cm², inducing transmembrane potential shifts of several to tens of millivolts. Superimposed on a –70 mV resting potential, this depolarization reaches the –40 to –30 mV activation threshold of L-type voltage-gated calcium channel (VGCC). Stimulation frequency dictates the subsequent calcium response. Low frequencies (<1 Hz) permit complete channel recovery between pulses, generating discrete Ca^2+^ transients. At 10–50 Hz, which is common for these scaffolds, channels cannot fully recover. The available channel pool declines across pulses, driving a summated, plateau-like Ca^2+^ elevation. Following VGCC-mediated influx, Ca^2+^ locally activates CaMKII at the channel pore and globally activates calcineurin via calmodulin. Calcineurin dephosphorylates NFAT to drive its nuclear translocation, while CaMKII phosphorylates CREB at activity-dependent promoters. These pathways initiate the transcription of immediate-early genes (c-fos, Arc) within the first hour, triggering BDNF and neurotrophic factor expression over subsequent hours. The temporal pattern of Ca^2+^ entry dictates the scale of this transcriptional cascade. Summated Ca^2+^ elevations drive stronger CREB phosphorylation and higher BDNF output compared to discrete transients. The central bioengineering challenge is balancing high charge injection capacity with long-term biocompatibility, motivating the development of fully bioresorbable conductive networks that degrade without adverse effects upon functional circuit restoration.

#### Applications of conductive biomaterials in central nervous system injury

CNTs and graphene derivatives provide high electrical conductivity combined with mechanical robustness, making them well-suited for neural interfacing in the CNS (Table 1). In TBI models, highly transparent (>85%), stretchable CNT microelectrode arrays enabled simultaneous optogenetic stimulation and high-resolution Ca^2+^ imaging, revealing trauma-induced intracellular Ca^2+^ hyper-influx in cortical neurons [150]. In SCI, aligned CNT/gelatin methacryloyl (GelMA) fibers synergized with DC stimulation to provide anisotropic guidance for neural stem cell (NSC) elongation (NF^+^, Tuj1^+^), achieving a Basso-Beattie-Bresnahan (BBB) score of 7.7 [158]. Similarly, integrating CNT-gelatin hydrogels with electroacupuncture successfully promoted endogenous NSC differentiation and restored neurogenic bladder function [159]. For macro-defects, 3D porous rGO scaffolds supported robust axonal ingrowth from brainstem nuclei [211, 165], while dielectrophoretically aligned rGO-coated PLGA microcapsules releasing NT-3 established spatiotemporal gradients that minimized astrogliosis, achieving an accelerated BBB score of 16 at 35 days post-injury [164]. Carbon nanomaterials offer an unmatched combination of electrical conductivity and anisotropic topographical guidance. However, their hydrophobicity and bioinertness render them intrinsically incompatible with the CNS parenchymal environment, where their indefinite persistence drives a sustained foreign-body cascade that progressively undermines the regenerative niche they are designed to support. Bridging this fundamental contradiction demands either the development of enzymatically cleavable CNT derivatives or the engineering of oxidation-labile graphene formulations whose degradation kinetics are matched to the chronology of axonal circuit restoration.

**Table 1:** Applications of conductive and piezoelectric materials in nerve regeneration.

Injury model	Electroactive category	Electroactive component	Platform design	Electrical properties	Regenerative outcomes	Reference
TBI	Conductive	CNTs	Film	Impedance: 0.20 ± 0.03 MΩ at 1 kHz	Increases intracellular Ca^2+^ influx.	[150]
TBI	Conductive	MXene (Ti_3_C₂T_x_) nanofilm	Hydrogel	Conductivity: 2–4 S/m	Promotes neuronal differentiation (Tuj1^+^↑) and inhibits astrocyte differentiation (GFAP↓) under ES (300 mV/cm).	[151]
TBI	Piezoelectric (Magnetoelectric)	Au nanostrip	Nanostrip	Induced voltage: 2.1 mV; current: 32.3 µA (at 300 rpm magnetic field)	Promotes neuronal differentiation (Tuj1, MAP2↑) and induces A2 neuroprotective astrocytes (S100A10↑, C3↓); activates PI3K‑Akt‑mTOR, inhibits NF‑κB; enhances synaptogenesis.	[152]
TBI	Piezoelectric (US-responsive)	BTO nanowires	Nanowire	d33 ≈ 100 pm/V; Induced voltage: 2 mV under US (1 MHz)	Accelerates neuronal differentiation; promotes synapse formation (SYP, GAP43↑); triggers rapid Ca^2+^ influx via neurotransmitters.	[153]
TBI	Piezoelectric (US-responsive)	BTO/rGO hybrid	Nanosticker	Induced voltage: 1 mV under US (1 MHz, 0.4 W/cm²)	Activates VGCC and increases Ca^2+^ influx, activates CaMKII-CREB pathway; upregulates BDNF; neuronal differentiation (Tuj1^+^↑) without ROS generation; enhances synaptogenesis (PSD95, SYP↑).	[154]
TBI	Piezoelectric (US-responsive)	CPLLA (metastable α′ crystal)	Fiber patch	d33 ≈ 44 pm/V; Output voltage: 0.2 V (1 MHz, 0.3 W/cm² US)	Activates Wnt pathway; promotes endogenous NSC differentiation into neurons; increases neuron/astrocyte ratio by 9.6-fold; modulates immune microenvironment (M2 microglia).	[155]
TBI	Piezoelectric (Magnetoelectric)	Graphene nanosheets	Nanopatch	Induced voltage: 1.8 mV (at 600 rpm magnetic field)	Activates MAPK/ERK pathway; promotes differentiation into functional neurons with voltage‑gated channels.	[156]
TBI	Piezoelectric (US-responsive)	PDA-modified nanocellulose	Hydrogel	d33 ≈ 4.8 pC/N; Output voltage: 1.5 V (under 400 W US)	Activates PI3K‑Akt pathway; promotes glutamatergic (VGLUT1^+^) and GABAergic (VGAT^+^) neuronal differentiation; PDA scavenges ROS and induces M2 microglia (IL‑10, Arg‑1↑).	[157]
SCI	Conductive	CNTs	Hydrogel fiber	Conductivity: 1.57 × 10^−^^4^ S/cm	Promotes axonal regeneration (NF^+^, Tuj1^+^↑), remyelination (MBP^+^↑) and reduces inflammation (Iba-1, CS56↓).	[158]
SCI	Conductive	CNTs	Hydrogel	Conductivity: 0.36 S/m	Promotes axonal regeneration (NF^+^, MBP^+^↑), reduces glial scar (GFAP, CS-56↓) and improves bladder function.	[159]
SCI	Conductive	MXene nanofilm	3D porous foam	Conductivity ≈ 20 S/m	Modulates macrophage polarization (M1→M2), reduces ROS (ROS↓, Nrf2↑), promotes neuronal differentiation (Tuj1, NeuN↑), activates VGCC and enhances synaptogenesis (PSD95↑, SYP-1↑).	[160]
SCI	Conductive	PPy/TA hydrogel	Hydrogel	Conductivity: 0.05–0.18 S/cm; Impedance: 10–17 Ω at 300 Hz	Activates endogenous NSC differentiation (Tuj1↑) and inhibits astrocyte differentiation (GFAP↓).	[161]
SCI	Conductive	PPy NPs	Hydrogel	Conductivity: 0.023–0.189 S/m	Protects BMSCs from ROS; promotes neurogenesis (Tuj1^+^, NeuN^+^↑) and remyelination (MBP^+^↑) via L-VGCC and PI3K/Akt/CREB pathways under ES *in vivo*.	[162]
SCI	Conductive	PPy NPs	Hydrogel	Conductivity: 0.002 S/cm	Activates L-VGCC, increases intracellular Ca^2+^ (CaMKII, CREB↑); promotes endogenous neurogenesis (Nestin^+^, NeuN^+^↑), reduces glial scar (GFAP, CSPGs↓).	[163]
SCI	Conductive	rGO/PLGA microcapsules	Hydrogel	N/A	Activates endogenous NSC differentiation (Tuj1^+^, MAP2^+^↑) and inhibits astrocyte differentiation (GFAP↓).	[164]
SCI	Conductive	rGO	3D porous foam	Sheet resistance: 0.55 ± 0.10 kΩ/sq; Conductivity: 0.47 ± 0.08 S/m	Promotes axonal regrowth (NF^+^, Tuj1^+^↑) and angiogenesis (RECA-1^+^↑) within the scaffold.	[165]
SCI	Conductive	Wet collagen	Hydrogel fiber	N/A	Promotes remyelination (MBP^+^, NF200^+^↑), improves axonal conduction and motor coordination.	[166]
SCI	Conductive	Wet collagen with NSCs	Hydrogel	N/A (External ES: 20 mA, 4 kHz)	Promotes glutamatergic neuron differentiation (VGlutT1↑) in graft; activates lumbar motor neurons and CPG circuits (c-Fos^+^↑); enhances NMJ innervation (SYP^+^/α-BTX^+^).	[167]
SCI	Piezoelectric (Magnetoelectric)	BTO-modified PVDF	Film	Output voltage: 2.9 V; current: 59 µA	Promotes neuronal differentiation (Tuj1, MAP2↑); activates VGCC; promotes CST axonal regeneration and motor neuron connectivity; restores motor evoked potentials and Ca^2+^ signaling.	[168]
SCI	Piezoelectric (Magnetoelectric)	Fe_3_O_2_@BTO NPs	Hydrogel	d33 ≈ 28 pm/V; Induced potential 8 µV (13 mT magnetic field)	Activates L-VGCC, increases intracellular Ca^2+^, promotes axonal regeneration (NF^+^↑), remyelination (MBP^+^↑) and synaptogenesis (SYN1^+^↑).	[169]
SCI	Piezoelectric (Magnetoelectric)	MXene (Ti_3_C₂T_x_) nanofilm	Film	Induced current: 7 µA (peak) at 200 rpm magnetic field (RMS: 4.9 µA)	Increases intracellular Ca^2+^ influx, promotes neuronal regeneration (Tuj1^+^, MAP-2^+^↑) and reduces lesion cavity.	[170]
SCI	Piezoelectric (US-responsive)	KNN nanowires	Nanofiber	d33 ≈ 20 pm/V; Output voltage: 12 V, current: 20.8 µA under US (150 kPa)	Promotes endogenous neurogenesis (Nestin^+^↑), angiogenesis (CD31^+^, VEGF^+^↑) and axonal regeneration (NF^+^, MBP^+^↑).	[171]
PNI	Conductive	Aniline Pentamer (AP)	Conduit	Conductivity: 3.89 × 10^−^^6^ S/cm	Modulates VGCC and activates MAPK (MEK/ERK) pathway, promoting SC maturation, myelin sheath formation and axonal regeneration.	[172]
PNI	Conductive	Aniline Trimer	Conduit	N/A	Upregulates Rap1 and mTOR pathways; ES enhances SC myelination and axonal elongation via these pathways.	[173]
PNI	Conductive	CNTs	Hydrogel	Charge storage capacity: 3.04 μF	ES promotes DRG axon outgrowth, SC migration and axon myelination (MBP↑); activates ERK2/AKT1 pathways.	[174]
PNI	Conductive	CNTs	Hydrogel	Conductivity: 3.1 ± 0.6 S/m	Promotes M2 macrophage polarization; reduces inflammation (TNF-α, IL-6); enhances SC migration and neurotrophic secretion.	[175]
PNI	Conductive	CNTs	Conduit	Conductivity: 3.90 × 10^−^^4^ S/cm	Supports SC growth; promotes myelination (MBP^+^) and axonal regeneration *in vivo*.	[176]
PNI	Conductive	CNTs	Nanotube	N/A	Promotes axonal regeneration and myelination; upregulates S100 and neurofilament expression.	[177]
PNI	Conductive	CNTs	Fiber	Conductivity: 3.52 × 10^−^^6^ S/cm	Promotes hADMSC differentiation into myelinating SC-like cells via c-Jun and Raf/ERK pathways; upregulates myelination genes (MBP, MPZ).	[178]
PNI	Conductive	Au nanorods	3D porous scaffold	Resistance: 1 × 10^8^ Ω	Promotes PC12 cell adhesion, proliferation and neural-specific protein expression (Nestin).	[179]
PNI	Conductive	Graphene	Conduit	Conductivity: 8.92 × 10^−^³ S/cm	Enhances SC proliferation, adhesion (Vinculin, N-cadherin) and neural gene expression (GFAP, Tuj1, NGF, BDNF).	[180]
PNI	Conductive	Graphene foam	3D porous scaffold	Resistance: 50 Ω	Activates MAPK pathway, promoting transdifferentiation into SC-like phenotypes (upregulation of S100, p75, MBP and NGF/BDNF secretion).	[181]
PNI	Conductive	Graphene nanosheets	Conduit	Conductivity: 0.0035 ± 0.0004 S/m	Promotes directional SC migration and elongation; upregulates Sox-10, MBP, NGF.	[182]
PNI	Conductive	GO, rGO	Conduit	N/A	Promotes SC myelination genes (Krox20, PMP22, NGF); upregulates S-100β, NF-200.	[183]
PNI	Conductive	Conductive Chitosan	Conduit	Ionic Conductivity: 133.23 ± 4.22 mS/cm	Combination of 4-AP and ES prolongs depolarization, increases neurotransmitter release, upregulates TrkB/Akt signaling and enhances BDNF/NGF, promoting regeneration/remyelination.	[184]
PNI	Conductive	Ionogel Battery (CS/Chondroitin Sulfate/HEC)	Hydrogel conduit	Output Voltage: 275 mV, Current: 475.7 μA (peak)	Modulates membrane potential and VGCCs, activating HIF-1α, VEGF, BDNF and GDNF expression.	[185]
PNI	Conductive	Mg wire coil	Hydrogel conduit	Output Voltage: 50 mV (alternating magnetic field: 0.5 mT, 50 Hz)	Reduces ROS, regulates inflammation, promotes angiogenesis (VEGF) and maintains mitochondrial ATP. ES upregulates BDNF and NGF.	[186]
PNI	Conductive	Mg (anode), FeMn (cathode)	Conduit	Voc: 0.98 V; Simulated electric field: 20–250 mV/mm	Promotes DRG neuron Ca^2+^ activity and neurite outgrowth; enhances SC proliferation and BDNF/CNTF/NGF/VEGF secretion.	[187]
PNI	Conductive	Mo, Mg	N/A	N/A	Prevents denervation muscle atrophy by distal nerve stump stimulation; maintains muscle fiber area and NMJ integrity.	[188]
PNI	Conductive	MWCNTs	Nanofibers	Conductivity: 22.67 ± 4.28 mS/cm	Promotes PC12 differentiation and neurite outgrowth; upregulates Tuj1, MAP2, ChAT.	[189]
PNI	Conductive	MWCNTs	Scaffold	Conductivity: 0.0006 S/m	Reduces ROS and promotes neuronal differentiation and neurite outgrowth, potentially via MAPK pathway.	[190]
PNI	Conductive	MXene (Ti_3_C₂T_x_)	Hydrogel conduit	Conductivity: 8.5 × 10^−^^4^ S/cm	Upregulates RANKL, activating NF-κB signaling to drive SC differentiation into myelinating subtypes.	[191]
PNI	Conductive	N-type Si membranes	Conduit	Resistivity: 0.06–0.09 Ω·cm	Enhances DRG Ca^2+^ activity; upregulates SC neurotrophic factors.	[192]
PNI	Conductive	PEDOT	Hydrogel conduit	Conductivity: 0.59 ± 0.02 S/cm	Under ES, promotes SC (S100) and neuronal (NF200) marker expression, promotes M2 macrophage polarization, reducing inflammation.	[193]
PNI	Conductive	PEDOT: PSS	Conduit	Conductivity: 0.07 S/cm (parallel), 0.02 S/cm (perpendicular)	Promotes directional SC and PC12 growth; upregulates neural markers.	[194]
PNI	Conductive	PEDOT: PSS	Hydrogel	Low impedance (1–10,000 Hz); CSC: 51 mC/cm²	Short-term (30 min) ES accelerates axonal growth and functional recovery.	[195]
PNI	Conductive	PEDOT: PSS	Hydrogel nanofiber	Conductivity: 65.3 S/cm	Activates mechanosensitive Piezo channels (Piezo-1/2) and integrin-β1/FAK/AKT pathway; increases Ca^2+^ influx; upregulates Atp2a2. Promotes neuronal differentiation, migration and M2 polarization.	[196]
PNI	Conductive	PEDOT: PSS	Hydrogel	Conductivity: 7.57 ± 0.46 × 10^−^^4^ S/cm	Facilitates SC migration, axonal growth and NMJ re-establishment, improving motor recovery and reducing muscle atrophy.	[197]
PNI	Conductive	PANI	Conduit	Conductivity: 1.7 S/m	ES upregulates endogenous BDNF, promoting SC proliferation and myelination (MBP).	[198]
PNI	Conductive	PANI	Hydrogel	Resistivity: 0.2–0.3 Ω·m	Restores bioelectrical signal transmission in transected nerves; promotes muscle contraction and functional recovery.	[199]
PNI	Conductive	PPy	3D porous scaffold	N/A (External ES: 20 Hz, 4 V, 0.1 ms pulses)	Promotes motoneuron regeneration; increases Fluorogold-labeled motoneurons; accelerates axonal outgrowth.	[200]
PNI	Conductive	PPy	Conduit	Conductivity: 15.56 mS/cm	Enhances nerve conduction velocity and myelinated fiber density *in vivo*.	[201]
PNI	Conductive	PPy	3D scaffold	Conductivity: 0.114 mS/mm	Enhances SC proliferation, migration and neurotrophic factors (BDNF, NGF); activates MAPK (ERK, p38) pathway *in vivo*.	[202]
PNI	Conductive	PPy	Hydrogel	N/A	Activates IL-17 signaling; promotes SC myelination (MBP, Sox-10, S100-β).	[203]
PNI	Conductive	PPy	Hydrogel	Conductivity: 148.9 ± 3.4 mS/m	Facilitates electrical signal transmission, promoting axonal growth and SC activity via enhanced Ca^2+^ influx and cAMP/PKA cascade.	[204]
PNI	Conductive	PPy	Nanofibers	N/A	Activates Ca^2+^/p-CREB/BDNF pathway in SCs; phosphorylates TrkB and Akt in PC12 cells. Promotes neurite outgrowth via BDNF.	[205]
PNI	Conductive	PPy	3D scaffold	Conductivity: 797 S/cm	Promotes directional neurite outgrowth, activates voltage-gated ion channels and MAPK/ERK cascades under ES.	[206]
PNI	Conductive	PPy	Hydrogel	Conductivity: 1.1 × 10^−^² S/cm	Activates pathways (e.g. PI3K/Akt) related to SC proliferation and differentiation, promoting S100 expression and myelination.	[207]
PNI	Conductive	PPy NPs	Conduit	Output voltage: 2.87 V	Activates insulin signaling (p-IRS1, p-ERK, p-p38, p-CREB), promoting SC and neuronal survival, reducing apoptosis and upregulating neurodevelopmental genes (Nanog, Pax3, Egr2).	[208]
PNI	Conductive	rGO	Nanofibrous scaffold	Conductivity: up to 4.05 × 10^−^² S/m	Promotes SC migration, proliferation and myelination (NGF, PMP22, Krox20); induces PC12 differentiation under ES.	[209]
PNI	Conductive	rGO	Nanofiber	Conductivity: 0.0443 ± 0.0004 S/m	Synergizes with ES to promote PC12 differentiation; upregulates Tuj1, MAP1b.	[210]
PNI	Conductive	rGO	Fiber	Conductivity: 329.4 S/m	Upregulates GAP-43, BDNF, TrkB, VEGF, α-SMA.	[211]
PNI	Conductive	rGO	Hydrogel	Conductivity: 2.66 ± 0.32 mS/cm	Enhances electrical transmission between nerve stumps, promotes SC migration and axonal growth, potentially via VGCC activation.	[212]
PNI	Conductive	rGO/PANI	Scaffold	Conductivity: 0.62 × 10^−^³ to 1.72 × 10^−^³ S/cm	N/A	[213]
PNI	Conductive	SiP nanosheets	Hydrogel	Conductivity: 0.25 S/m	Promotes M2 polarization; enhances angiogenesis (HIF-1α, VEGF); induces MSC-to-SC differentiation.	[214]
PNI	Conductive	SA/CS ionic hydrogel	Hydrogel fiber	N/A	Enhances endogenous electric fields, promoting MSC proliferation, differentiation and directional migration toward a neuronal lineage.	[215]
PNI	Conductive	Tetra(aniline)	Hydrogel fiber	Conductivity: 6.97 × 10^−^^6^ S/cm	Promotes PC12 cell neurite outgrowth and differentiation; upregulates ERK1/2 phosphorylation.	[216]
PNI	Conductive	MXene nanosheet	Conduit	Conductivity: 233 ± 12.49 μS/cm	Guides directional SC migration; promotes axonal regeneration and myelination.	[217]
PNI	Conductive	Zn (anode), Mo (cathode)	Conduit	Vout: 0.5 V (constant current discharge 25 μA/cm²)	Promotes SC proliferation and ATP production; accelerates axonal growth and myelination.	[218]
PNI	Piezoelectric (Friction-responsive)	BTO	Scaffold	Voc: 35–40 mV	Upregulates MBP, MPZ, TRPC2, YAP, TAZ genes.	[219]
PNI	Piezoelectric (US-responsive)	PDA-coated BTO NPs	Conduit	Conductivity: 2.6 × 10^−^^4^ S/cm; Output voltage: 0.078 V under 0.3 W/cm² US	Promotes M2 macrophage polarization and PC12 axon elongation, activates Ca^2+^ channels and cAMP-dependent pathway.	[220]
PNI	Piezoelectric (US-responsive)	BTO NPs	Hydrogel	Output Voltage: 0.5 V under US	Upregulates PIEZO1 (astrocytes) and PIEZO2 (SCs); increases Ca^2+^ influx; activates ATP synthase and MFN/OPA1-mediated mitochondrial fusion; activates PIEZO-Ca^2+^-CREB axis.	[221]
PNI	Piezoelectric (Shockwave-responsive)	PVDF-TrFE	Conduit	Voc: 200 mV (4 bar shockwave)	Upregulates Tuj1, MAP2, ChAT (neurons) and Krox20, PMP22 (SCs).	[222]
PNI	Piezoelectric (US-responsive)	PLLA	Conduit	Output voltage: 161.2 mVpp under US	Activates VGCCs and cAMP/PKA pathway, upregulating GAP-43 and promoting axon elongation.	[223]
PNI	Cond/Piezo (Friction-responsive)	rGO/PLLA	Conduit	N/A	Activates VGCCs and promotes SC activity and neurite outgrowth.	[224]
PNI	Cond/Piezo (US-responsive)	rGO/PVDF	Conduit	d33: 17.4 ± 0.79 pm/V; Output voltage: 0.5 V under US	Reduces ROS and apoptosis; activates voltage-gated channels and promotes nerve regeneration.	[225]
PNI	Piezoelectric (Magnetoelectric)	Ag nanoflakes	Conduit	Conductivity: 25.48 ± 3.77 S/cm	Activates taurine/hypotaurine metabolism; upregulates ADO and FMO1 enzymes; enhances intercellular Ca^2+^ influx.	[226]
PNI	Tribo-piezoelectric (Friction-responsive)	TENG/PENG hybrid	Conduit	Voc: 501 ± 8 V; Power density: 72 mW/m²	Regulates SC aggregation, proliferation and myelination; promotes axonal regeneration.	[227]
PNI	Tribo-piezoelectric (US-responsive)	ACT-TENG, Mg electrodes	N/A	Voc: 7.8 V (40 MΩ); Isc: 60.0 μA (1 Ω); Power density: 102.4 μW/cm²	Enhances axonal myelination; improves nerve conduction velocity.	[228]
PNI	Piezoelectric (Friction-responsive)	ZnO NPs	Hydrogel conduit	d33 > 20 pm/V; Output voltage: 0.12 V	Activates NCAM and GAP43, promotes SC proliferation and directional migration (possibly via PI3K/AKT-Nrf2 pathway).	[229]
PNI	Piezoelectric (US-responsive)	ZnO NPs	Conduit	Piezoelectric coefficient: 12 pm/V	Promotes SC proliferation (Ki67), neural markers (S100, MBP, Tuj1, NGF) and angiogenesis (VEGF).	[230]
PNI	Piezoelectric (Friction-responsive)	ZnO NPs, rGO	Conduit	Conductivity: 1.08 × 10^−^³ S/cm; Voc: 4.6 V; Isc: 1.9 μA	Provides ES to denervated muscles to inhibit atrophy; guides SC migration.	[231]
PNI	Cond/Piezo (Friction-responsive)	CNTs@GelMA/PLLA	Conduit	Output voltage: 5.96 V; Conductivity: 0.3 S/m	Promotes SC elongation, DRG neurite outgrowth, myelination and motor function recovery.	[232]

4-AP, 4-aminopyridine; Ag, silver; ATP, adenosine triphosphate; Au, gold; BDNF, brain-derived neurotrophic factor; BMSC, bone marrow mesenchymal stem cell; BTO, barium titanate; Ca^2+^, calcium ion; ChAT, choline acetyltransferase; CNTF, ciliary neurotrophic factor; CNTs, carbon nanotubes; CPG, central pattern generator; CPLLA, crystallized poly(L-lactic acid); CS, chitosan; CSC, charge storage capacity; CST, corticospinal tract; d33, piezoelectric charge coefficient; DRG, dorsal root ganglion; ES, electrical stimulation; GAP43, growth associated protein 43; GDNF, glial cell line-derived neurotrophic factor; GelMA, gelatin methacryloyl; GFAP, glial fibrillary acidic protein; GO, graphene oxide; hADMSC, human adipose-derived mesenchymal stem cell; HEC, hydroxyethyl cellulose; Isc, short-circuit current; KNN, potassium sodium niobate; MAP2, microtubule-associated protein 2; MBP, myelin basic protein; Mg, magnesium; Mo, molybdenum; MPZ, myelin protein zero; MSC, mesenchymal stem cell; MWCNTs, multi-walled carbon nanotubes; NCAM, neural cell adhesion molecule; NeuN, neuronal nuclei; NF/NF200, neurofilament; NGF, nerve growth factor; NMJ, neuromuscular junction; NPs, nanoparticles; NSC, neural stem cell; PANI, polyaniline; PDA, polydopamine; PEDOT, poly(3,4-ethylenedioxythiophene); PEDOT, PSS, poly(3,4-ethylenedioxythiophene) polystyrene sulfonate; PENG, piezoelectric nanogenerator; PLLA, poly-L-lactic acid; PNI, peripheral nerve injury; PPy, polypyrrole; PSD95, postsynaptic density protein 95; PVDF, polyvinylidene fluoride; PVDF-TrFE, poly(vinylidene fluoride-trifluoroethylene); rGO, reduced graphene oxide; RMS, root mean square; ROS, reactive oxygen species; SA, sodium alginate; SC, Schwann cell; SCI, spinal cord injury; SYP, synaptophysin; TA, tannic acid; TBI, traumatic brain injury; TENG, triboelectric nanogenerator; Tuj1, beta-III tubulin; US, ultrasound; VEGF, vascular endothelial growth factor; VGCC/L-VGCC, voltage-gated calcium channels/L-type voltage-gated calcium channels; Voc, open-circuit voltage; ZnO, zinc oxide.

MXenes (two-dimensional transition metal carbides) combine metallic-grade conductivity with intrinsic surface hydrophilicity, offering a distinct advantage for biofluid-compatible neural interfaces [233]. An injectable silk fibroin/MXene hydrogel for TBI robustly skewed NSC differentiation toward mature neurons while suppressing astrocytic lineage commitment, markedly contracting the lesion cavity [151]. For severe SCI, a 3D PLGA/MXene scaffold co-formulated with Mg(OH)₂, brain-derived extracellular matrix (ECM) and neural progenitor cells (NPCs) orchestrated a multimodal microenvironmental rescue. This platform attenuated oxidative stress and modulated VGCC activity to sustain concurrent axonal regeneration and remyelination [160]. MXenes offer superior aqueous compatibility and charge-injection capacity. However, their susceptibility to oxidative and hydrolytic degradation in physiological fluids leads to progressive conductivity loss. Stabilization strategies, such as antioxidative surface passivation and encapsulation within ion-selective elastomeric matrices, are therefore not optional refinements but essential prerequisites for clinically viable MXene-based neural constructs.

PPy-based conductive hydrogels uniquely address the mechanical mismatch between rigid electrodes and soft neural tissue, enabling conformal contact with irregular lesion geometries. Soft PPy-based hydrogels doped with tannic acid minimized post-traumatic SCI cavitation and promoted NSC fate, yielding a BBB score of 13 at 6 weeks [161]. Furthermore, injectable supramolecular agarose/gelatin/PPy hydrogels allowed conformal filling of irregular lesion cavities, locally upregulating Ca^2+^-dependent signaling cascades [163]. Addressing the acute oxidative trauma in CNS injuries, dual-functional PPy/collagen/hyaluronic acid matrices scavenged ROS, shielding encapsulated bone marrow-derived mesenchymal stem cells from apoptosis [162]. The primary limitations of CP hydrogels are dopant leaching and progressive embrittlement of the polymer backbone *in vivo*. Resolving these limitations demands a departure from conventional doping strategies toward molecularly integrated, biodegradable conjugated backbones in which electroactivity and structural integrity are encoded within the same degradable repeat unit.

Natural ECM-derived scaffolds provide unmatched biological recognition and low immunogenicity, serving as ideal delivery vehicles for therapeutic biologics and cells in CNS repair. A collagen fiber-hydrogel delivering microRNA-219/338 directed endogenous oligodendrocyte precursor cells toward mature, myelinating phenotypes, significantly recovering sensory function [166]. Combinatorially, transplanting stem cell-derived spinal cord-like tissue within a biomimetic collagen matrix, supplemented with non-invasive tail nerve ES, accelerated graft maturation into glutamatergic neurons and successfully reactivated dormant lumbar CPGs [167]. Natural polymers provide exceptional bio-recognition and negligible immunogenicity, serving as ideal vehicles for biologics and cells. However, the intrinsic electrical insulation of natural polymers necessitates reliance on tethered external ES, which limits spatial uniformity of charge delivery and introduces risks of localized Joule heating. The field should progress toward bio-hybrid architectures that integrate wireless piezoelectric nanogenerators or embedded conductive traces into natural polymer matrices, enabling untethered, physiologically responsive neuromodulation.

Collectively, these platforms converge upon shared mechanisms: (i) providing a permissive bridge for directional axonal ingrowth; (ii) facilitating bioelectric current propagation to activate VGCCs and downstream Ca^2+^-dependent cascades; (iii) remodeling the neuroinflammatory microenvironment toward a pro-regenerative M2 phenotype; and (iv) promoting remyelination. The consistent superiority of combined material–ES approaches over either intervention alone underscores the therapeutic potential of integrated ‘regenerative rehabilitation’ strategies. Despite these promising preclinical milestones, a significant translational gap remains between rodent models and clinical reality. The complex pathophysiology of human CNS injury demands electroactive scaffolds that degrade synchronously with tissue neogenesis. Validation in large-animal models is a necessary prerequisite for establishing the long-term biocompatibility and electrophysiological stability of these platforms over extended rehabilitation timescales.

#### Applications of conductive biomaterials in peripheral nerve injury

CNT-integrated conduits offer a unique combination of high electrical conductivity and fibrillar topography that closely mimics native extracellular matrix architecture, providing both electro-conductive and contact guidance cues for peripheral nerve repair. Injectable nanohybrid hydrogels encapsulating pristine CNTs accelerated DRG neurite outgrowth and SCs myelination via the ERK2/AKT1 signaling pathways [174], while endoscopic adhesive CNT-hydrogels orchestrated an immuno-regenerative microenvironment specifically for cavernous nerve restoration [175]. For macroscopic injuries, CNT/sericin conduits coupled with external ES [176] and aligned CNT yarns [177] successfully bridged critical 10-mm and 15-mm defects, achieving autograft-equivalent regenerative performance. Additionally, electrospun poly(p-dioxanone)/CNT nanofiber yarns synergistically directed the transdifferentiation of mesenchymal stem cells (MSCs) into SC-like lineages [178]. Aligned coaxial nanofibers with a conductive multi-walled CNT (MWCNT) shell and a core co-releasing nerve growth factor (NGF) and Lycium barbarum polysaccharides significantly upregulated Tuj1, Map2, and Chat expression [189]. Electrospun PCL–collagen scaffolds incorporating MWCNTs and Bacopa monnieri extract reduced oxidative stress and promoted neuronal differentiation via the MAPK pathway in sciatic crush models [190]. CNTs offer unparalleled electrical conductivity and a high-aspect-ratio topography that perfectly mimics native extracellular matrix fibers, driving rapid SC migration. The principal translational concern is their resistance to biodegradation and tendency to aggregate *in vivo*, which may cause chronic nerve compression or granuloma formation. Translation will require the development of biocleavable CNT derivatives or reversible functionalization strategies that guarantee complete clearance once the nerve gap has been bridged.

Graphene derivatives provide a two-dimensional planar architecture with high specific surface area, well-suited for micropatterning and localized charge injection to guide SC behavior in peripheral nerve repair. A three-dimensional-printed multilayer graphene/PCL conduit functionalized with polydopamine (PDA)/RGD enhanced focal adhesion assembly and SC proliferation across a 15-mm gap [180]. Under localized ES, 3D graphene foams activated the MAPK pathway to achieve a near 90% SC transdifferentiation efficiency [181]. Diverse structural formulations, including micropatterned PLCL/graphene nanosheets [182], thermally stable PLMC/gelatin/rGO matrices [183], rGO-coated meshes [209], rGO-decorated PCL nanofibril fillers [210], longitudinally microchanneled rGO fibers [211] and pluronic-coated rGO hydrogel filler [212], have consistently demonstrated robust electrotransduction that upregulates key myelination markers and supports structural nerve regeneration. The mechanical brittleness of graphene and the tendency of nanosheets to restack via π–π interactions *in vivo* limit their compliance with the dynamic mechanical environment of peripheral nerves. Future designs should anchor low concentrations of rGO monolayers onto elastomeric, bioresorbable polymer backbones to prevent mechanical mismatch and fibrosis.

Conductive oligomers decouple electroactivity from permanent material biopersistence, offering a degradable alternative to bulk conjugated polymers for peripheral nerve applications. Electrospun PGCL/aniline pentamer conduits effectively modulated VGCC activity, promoting compact myelin assembly [172]. Similarly, gastrodin-functionalized polyurethane copolymers incorporating aniline trimers targeted the Rap1 and mTOR pathways, driving robust long-distance axonal regeneration [173]. Polyaniline (PANI)-based systems enable tunable bioelectrical interfaces for peripheral nerve repair. PANI-based platforms, including hydrogel conduits [198], polymeric conduit [198], silk-knitted scaffolds [213] and peptide nanofiber networks [216], have shown that substrate conductivity can drive SC proliferation and neurite outgrowth, in some cases outperforming exogenous neurotrophic factor delivery. Notably, a stretchable polyacrylamide/PANI hydrogel exploited near-infrared irradiation to dynamically modulate local conductivity, restoring compound muscle action potentials and gait [199].Conductive oligomers elegantly solve the chronic toxicity of bulk conjugated polymers by enabling controlled hydrolytic degradation. The primary limitation of this class is premature conductivity loss as the polymer matrix degrades, which may not align with the slow timeline of peripheral axonal regeneration in humans. Engineering block copolymers with tunable electrical attenuation kinetics synchronized to the spatial and temporal requirements of SC myelination represents a critical design objective.

PPy-based systems offer high charge injection density and electrochemical stability and have been formulated across a range of structural configurations to address diverse clinical requirements in peripheral nerve repair. Highly aligned PPy/silk fibroin scaffolds coupled with ES robustly stimulated axonal regeneration via the ERK and p38 MAPK cascades [202]. Similarly, PPy/Poly(D, L-lactide)(PDLLA) conductive conduits were fabricated to achieve functional recovery with ES comparable to autografts [201]. Addressing minimally invasive delivery, self-healing hyaluronic acid/PPy hydrogels activated the IL-17/IL-17RA axis to accelerate myelination [203], while sutureless PPy fibrous bandages effectively mitigated downstream denervation-induced muscle atrophy [204]. PPy has also been integrated into multi-modal architectures, including magnetic PCL/Fe_3_O_4_ nanofibers [205], 3D GelMA/chitosan double-networks [206] and self-powered, motion-harvesting conduits for on-demand drug release [207, 208]. Notably, aligned chitosan/PPy scaffolds demonstrated that even a brief 1-hour intraoperative ES session profoundly accelerates long-term axonal elongation [200]. Despite these advantages, bulk PPy is mechanically rigid and brittle, lacking the suturability required for clinical microsurgery. Progressive leaching of cytotoxic dopant ions remains an unresolved concern. Core–shell architectures or supramolecular encapsulation of PPy within soft biomimetic hydrogels could preserve surface conductivity while conferring the mechanical compliance necessary for clinical application.

PEDOT:PSS provides mixed electronic and ionic conductivity together with excellent aqueous processability, enabling fabrication of soft, conformal bio-interfaces that closely match the mechanical properties of peripheral nerve tissue. Highly aligned porous PLGA/PEDOT: PSS conduits delivered combined topographic and electrical guidance [194]. A nerve-mimetic adhesive hydrogel (CA-HE) with flexible PEDOT: PSS electrodes dramatically accelerated motor recovery via short-duration intraoperative ES [195]. At the molecular level, GelMA/tannic acid/PEDOT: PSS patches directly activated mechanosensitive Piezo channels and the integrin-β1/FAK/AKT signaling axis [196]. Injectable carboxymethyl chitosan [197] and CS-HEC@PEDOT [193] matrices further confirmed the efficacy of PEDOT in rescuing target muscle atrophy. The key translational limitation is the propensity of the PSS dopant to undergo phase separation and leaching *in vivo*, eliciting localized inflammatory responses. Developing PSS-free PEDOT formulations stabilized by biomacromolecular dopants (e.g. heparin, hyaluronic acid, or dextran sulfate) represents a promising route toward highly biocompatible, tissue-integrating electroceuticals.

Metallic components deliver the highest intrinsic conductivity among conductive biomaterial classes and uniquely enable the fabrication of self-powered, wireless galvanic neurostimulators that eliminate the need for percutaneous leads. Gold nanorods dispersed in silk fibroin enhanced PC12 cell adhesion and Nestin expression [179]. Pushing toward autonomous systems, fully biodegradable micro-batteries exploiting Mg–FeMn galvanic couples [187] or Zn–Mo interfaces [218] generated sustained endogenous electric fields utilizing physiological fluids as electrolytes, significantly accelerating motor recovery. Similarly, transient Mo/Mg electrodes acting as long-lived bioresorbable stimulators successfully prevented target muscle atrophy [188]. The primary limitation is the difficulty of controlling corrosion kinetics: rapid degradation of transient metals such as Mg and Zn generates localized hydrogen gas and alkalization, which can damage regenerating nerve tissue. Precision alloying and the application of ion-selective, pH-buffering nanocoatings are needed to regulate bioresorption rates and prevent localized metal-ion toxicity.

Emerging platforms exploit multi-dimensional signal transduction mechanisms that extend beyond conventional electronic conductivity. Conductive silk fibroin/MXene conduits upregulated RANKL to promote SC differentiation [191], while 4D-printed MXene/PLATMC conduits underwent thermally triggered self-curling at physiological temperatures to facilitate intraoperative application [217]. Ionically conductive chitosan conduits loaded with 4-aminopyridine (4-AP) prolonged axonal depolarization [184], eel-inspired ionogel batteries generated continuous ionic gradients to suppress inflammation [185] and oriented chitosan/PCL–sodium alginate composite scaffolds enhanced MSC differentiation toward neuronal lineages [215]. Furthermore, hydrogen-releasing conduits [186], bioresorbable N-type silicon membranes [192] and silicon phosphorus nanosheets [214] successfully leveraged alternative biophysical cues to promote neuroregeneration. Ionic hydrogels inevitably lose mobile charge carriers via passive diffusion into surrounding tissues, resulting in a progressive decline in effective conductivity. MXene-based systems face rapid oxidative degradation in physiological environments. Advancing ionotronic systems will require the design of semi-permeable elastomeric encapsulants that restrict ion leakage while maintaining electrochemical communication with the nerve, together with robust antioxidative surface functionalizations to stabilize MXene components over clinically relevant timescales.

### Piezoelectric biomaterials

Piezoelectric biomaterials transduce mechanical stress into electrical polarization, autonomously providing physiologically relevant electrical cues. Because neural tissues are highly responsive to biophysical stimuli, these materials can modulate cellular fate and support neuroregeneration without the limitations of conventional tethered electrodes [25, 27]. By harvesting ambient biomechanical energy or responding to non-invasive external triggers (e.g. ultrasound, magnetic fields), these platforms function as wireless bioelectric modulators at the lesion interface, directly addressing the electrophysiological deficits associated with neural injuries [26, 139]. These materials are primarily classified into inorganic piezoelectric nanomaterials, organic piezoelectric polymers and composite/hybrid systems.

#### Categories and generation mechanisms of piezoelectric materials

Inorganic piezoelectric nanomaterials, encompassing perovskite-type ceramics (e.g. BaTiO_3_, (K_0.5_Na_0.5_)NbO_3_ (KNN)) and wurtzite-structured oxides (ZnO), are distinguished by their high electromechanical coupling efficiencies. In tetragonal BaTiO_3_, spontaneous polarization originates from the off-center displacement of the Ti⁴^+^ ion relative to its O²^−^ octahedral cage [234]. However, their clinical translation as monolithic implants is substantially impeded by their intrinsic rigidity and concerns regarding heavy-metal toxicity (e.g. Pb^2+^ leakage), necessitating surface functionalization or elastomeric encapsulation [234, 235].

Organic piezoelectric polymers offer a mechanically compliant alternative with tunable biodegradation kinetics [139, 236]. Poly(vinylidene fluoride) (PVDF) and its copolymer P(VDF-TrFE) represent archetypal systems, wherein electroactivity is governed by the all-trans conformation of the polar β-phase [237, 238]. In contrast, poly(L-lactic acid) (PLLA) exhibits shear piezoelectricity (d_14_) arising from the stress-induced rotation of chiral carbonyl (C = O) dipoles, and requires no electrical poling [239]. Naturally derived biopolymers (e.g. collagen, cellulose) offer intrinsic biomimicry but possess modest piezoelectric coefficients (*dᵢ* < 10 pC N^−^¹), which limits their role to bioactive modifiers rather than primary electrical generators [28].

Piezoelectric composites and hybrid systems synergistically combine the high electrical output of ceramics with the mechanical compliance of polymers [240]. Ceramic nanoparticles frequently serve as nucleating agents to promote polar β-phase crystallization in PVDF matrices [238, 241]. Beyond solid-state mechanisms, magnetoelectric hybrids (e.g. Fe_3_O_4_@BaTiO_3_) couple magnetic and electrical domains, enabling remote stimulation under alternating magnetic fields by exploiting localized magnetostrictive deformation [169]. Mechanistically, under biomechanical loading or external triggers, these scaffolds generate transient surface charge densities [242]. The resulting electric potentials depolarize adjacent neural cell membranes, activating VGCCs and promoting localized Ca^2+^ influx. This initiates downstream signaling cascades, most notably the MAPK/ERK pathway, to regulate neural differentiation and network remodeling [243].

#### Applications of piezoelectric materials in central nervous system injury

In TBI, a key advantage of piezoelectric systems is their capacity for non-invasive transcranial activation, circumventing the need for surgical electrode implantation. Ultrasound (US)-responsive systems provide non-invasive transcranial activation. An electrospun metastable PLLA patch (d_33_ ≈ 44 pC N^−^¹) generated 0.2 V under low-intensity US, promoting endogenous NSC neurogenesis via the Wnt pathway and elevating the neuron-to-astrocyte ratio 9.6-fold [155]. At the single-cell level, BaTiO_3_ nanowires anchored onto NSC membranes generated localized potentials (≈ 2 mV) under US, activating mechanosensitive channels to accelerate functional differentiation [153]. To reduce nanoparticle-induced intracellular ROS, BTO/rGO nanocomposites confined the piezoelectric effect to the extracellular membrane, thereby promoting synaptogenesis without ROS accumulation [154]. For deeper or more inaccessible lesions, magnetic-responsive systems eliminate tissue acoustic attenuation; a living artificial dura mater integrated with gold nanostrips generated 2.1 mV under a rotating magnetic field, shifting reactive astrocytes toward the neuroprotective A2 phenotype while suppressing NF-κB signaling [152]. Extending this magnetic actuation strategy, conductive graphene nanosheets deployed as wireless nanoelectrode constructs to encapsulate NSCs generated 1.8 mV via strain-mediated induction under magnetic rotation, promoting neuronal specification (41% MAP2^+^) [156]. Addressing acute oxidative stress, a piezoelectric nanocellulose hydrogel coated with PDA achieved 1.5 V output under US while simultaneously scavenging ROS, remodeling the neuroimmune microenvironment [157]. US- and magnetically actuated piezoelectric platforms share a key advantage in TBI: the complete elimination of implanted hardware in a mechanically vulnerable, edematous parenchyma. Their principal limitation is the incomplete characterization of long-term inorganic component fate within the closed cranial cavity, and the unresolved constraint of transcranial acoustic attenuation. Future designs must prioritize fully bioresorbable compositions and validate stimulation dosimetry under clinically realistic skull geometries.

In SCI, piezoelectric scaffolds offer a distinct advantage in bridging macroscopic longitudinal tissue cavities while providing sustained directional electrical cues to overcome inhibitory glial scar environments. Biodegradable PLA scaffolds incorporating KNN nanowires generated a 12-V output under US, counteracting myelin-associated inhibitors to support robust axonal elongation (NF^+^) and functional angiogenesis (CD31^+^) [171]. Magnetoelectric hydrogels loaded with Fe_3_O_4_@BaTiO_3_ core–shell nanoparticles delivered low-amplitude 8 μV interfacial potentials, selectively activating L-type VGCCs to accelerate remyelination (MBP^+^) without inducing intracellular calcium overload [169]. Furthermore, a BaTiO_3_-modified PVDF piezoelectric nanocapacitor introduced a wireless charge-storage mechanism, releasing a sustained 2.9 V discharge over 2 hours following magnetic stimulation, recovering disrupted Ca^2+^ dynamics in caudal motor neurons and re-establishing motor evoked potentials [168]. Building on magnetic actuation, conductive MXene (Ti_3_C₂T_x_) nanofilms generated stable localized induction currents (7 μA) under a rotating magnetic field, promoting NSC differentiation into cholinergic neurons and restoring electrophysiological conduction in SCI models [170]. The outstanding challenge is the dependence on external US equipment, which limits suitability for long-term, unsupervised rehabilitation. In SCI, high-output piezoelectric and magnetoelectric scaffolds provide sustained wireless stimulation across large lesion cavities, a capability unattainable by passive conduits or tethered electrodes. However, the high voltages reported (up to 12 V) raise unresolved safety concerns regarding off-target excitation and electrochemical tissue damage. Establishing validated therapeutic stimulation windows, and resolving the mechanical mismatch between rigid inorganic components and the dynamic spinal environment, remain the central translational challenges.

Collectively, piezoelectric and magnetoelectric scaffolds share a common therapeutic mechanism in both TBI and SCI: wirelessly restoring physiological membrane potential and rescuing trauma-induced Ca^2+^ signaling deficits. The reported material output voltages, spanning from single-digit microvolts to double-digit volts, complicate a uniform mechanistic framing. At the cell-material interface, microvolt-level outputs fall significantly below typical VGCC gating thresholds. These low-magnitude signals more plausibly modulate local membrane capacitance or act synergistically with material deformation to engage mechanosensitive ion channels. Conversely, macroscopic volt-level outputs generate local electric fields that, through spatial attenuation and interface impedance, induce millivolt-scale transmembrane depolarizations. Provided the field gradients remain below electroporation thresholds, this attenuation safely recruits VGCCs and broader ionic conductances. Systematically mapping these macroscopic voltage ranges to their distinct channel targets and downstream cascades will provide a much-needed mechanistic basis for this structural divergence. However, their structural design and application strategies diverge considerably based on distinct pathological requirements. BaTiO_3_ exemplifies this divergence: in TBI, it is deployed as membrane-anchored nanostructures to enable targeted US activation while avoiding ROS accumulation in the edematous cranial cavity; in SCI, it is incorporated into bulk hydrogels or capacitive constructs to provide the sustained, high-voltage fields required to bridge large longitudinal gaps and overcome inhibitory glial scars. For US-driven TBI systems, the primary translational challenge is transcranial acoustic energy attenuation; for SCI, it is the mechanical mismatch and asynchronous degradation of inorganic components within the dynamic spinal environment. Future platforms should be fully bioresorbable and lead-free, with degradation kinetics matched to the timeline of CNS tissue regeneration.

#### Applications of piezoelectric materials in peripheral nerve injury

Unlike CNS applications, peripheral nerve repair demands conduits that can physically span large defects (≥10–15 mm) while delivering continuous electrical cues over extended periods to prevent irreversible target muscle atrophy, a requirement well-suited to piezoelectric systems capable of harvesting native biomechanical energy.

US-driven P(VDF-TrFE) aligned conduits generated 200 mV under non-invasive shockwaves, markedly upregulating SC myelination markers (Krox20, Pmp22) to repair a 15-mm sciatic nerve defect [222]. Similarly, an acousto-responsive PLLA/PEG conduit enhanced GAP-43 expression and directed growth cone propagation via the cAMP/PKA cascade [223]. To introduce multifunctionality, piezoelectric–conductive composite microcarriers (BTNP@PDA/CS) generated a 0.078 V output under localized US, polarizing the microenvironment toward anti-inflammatory M2 macrophage differentiation [220]. Toward extracellular matrix biomimicry, a BaTiO_3_/collagen hydrogel demonstrated that US-driven signals (about 0.5 V) preferentially activate mechanosensitive PIEZO1 in Schwann cells and PIEZO2 in peripheral neurons, enhancing ATP synthesis [221]. Relative to their CNS counterparts, US-driven piezoelectric systems operate with a fundamental anatomical advantage in the PNS: the absence of a bony acoustic barrier enables efficient energy delivery at lower intensities with minimal attenuation, improving both stimulation precision and safety. The outstanding challenge is the dependence on external US equipment, which limits suitability for long-term, unsupervised rehabilitation.

Harvesting native biomechanical energy offers an autonomous, activity-coupled stimulation paradigm particularly suited to peripheral nerve repair, eliminating the need for external power sources or tethered leads. A fully implantable triboelectric/piezoelectric hybrid nanogenerator, driven by host respiratory movement, delivered activity-regulated electrical cues that promoted SC migration and myelin sheath assembly [227]. Self-powered ZnO/PCL matrices and anisotropic ovalbumin/BaTiO_3_ scaffolds converted kinetic energy from treadmill exercise or mechanical friction into localized potentials (35–40 mV), upregulating mechanotransduction pathways (TRPC2, YAP/TAZ) and vascular endothelial growth factor (VEGF) secretion [230, 219]. A multi-dimensionally oriented GelMA/PEGDA/ZnO conduit fabricated via digital light processing (DLP) 3D printing generated 0.12 V under physiological stress, directing SC migration via the PI3K/AKT–Nrf2 pathway [229]. This paradigm is uniquely enabled by the mechanical dynamics of the peripheral anatomical environment and has no practical equivalent in the CNS, where parenchymal deformation is insufficient for reliable piezoelectric transduction. The principal limitation is the stochastic nature of patient movement, which introduces variability in stimulation dose. Integrating on-device rectification and charge-storage elements to buffer irregular mechanical inputs represents a critical design priority.

To optimize charge distribution and address local oxidative stress, hybrid conduits have been developed. A bilayer conduit featuring an inner ionically conductive chitosan/rGO layer and an outer piezoelectric PLLA shell combined tensile strength with mechanical piezoelectric responsiveness [224]. A bilayer conduit integrating an aligned piezoelectric PLLA shell with a conductive CNTs@GelMA inner hydrogel exploited routine body movements to generate surface electricity, enhancing SC excitability and successfully bridging a 10-mm sciatic nerve defect with concurrent remyelination and motor function recovery [232]. Inspired by bamboo’s hierarchical hollow architecture, a hybrid scaffold coupling a piezoelectric PVA/glycerol hydrogel shell doped with tannic acid-functionalized BaTiO_3_ nanoparticles, with an aligned PCL/CNT inner fiber layer generated a consistent 0.95 V output under physiological mechanical stress, autonomously activating VGCCs and the Notch/MAPK axis to drive neurovascularization and achieve autograft-comparable axonal regeneration [244]. A more complex design utilizing a microgrooved rGO/GelMA core and a PVDF/PLGA outer layer combined ROS scavenging with a 0.5 V output, reducing oxidative apoptosis [225]. Extending into magnetic and triboelectric modalities, magnetoelectric composite conduits (PCLG/AgNF) generated localized electrical pulses under oscillating magnetic fields, stimulating taurine metabolic pathways and restoring intracellular Ca^2+^ dynamics [226]. An on-demand bioresorbable neurostimulator powered by an US-driven triboelectric nanogenerator (ACT-TENG) utilizing transient Mg electrodes yielded 7.8 V to promote axonal myelination, undergoing controlled acoustic degradation post-therapy [228]. Similarly, a biomimetic microneedle conduit (rGO/ZnO) delivered a 4.6-V open-circuit voltage(Voc) to prevent target muscle atrophy while guiding SCs through inner microchannels [231]. Magnetoelectric and triboelectric scaffolds generate transient surface potentials from tens of millivolts to several volts, depending on particle composition and input amplitude. At the low end, the induced potential remains subthreshold for VGCC activation; observed Ca^2+^ responses likely originate from mechanosensitive channels. At the high end, the induced depolarization crosses the VGCC threshold. The pulsed nature of these inputs, driven by alternating fields or intermittent strain, allows channels to remain within their recovery window between events. This temporal pattern generates discrete Ca^2+^ transients, distinguishing it from the summated elevations seen under continuous high-frequency ES. Hybrid architectures address the intrinsic limitation of single-component systems by consolidating electrical, mechanical and antioxidative functions within a single implant. The tubular conduit geometry of peripheral nerve repair constrains charge delivery toward the regenerating stump, conferring a spatial specificity difficult to achieve in the geometrically irregular CNS lesion environment. The outstanding challenges are interfacial delamination under cyclic mechanical loading and the controlled degradation of transient metallic components. Monolithic fabrication via gradient electrospinning or multi-material additive manufacturing represents the most promising route to mechanically robust, multifunctional peripheral piezoelectric conduits.

Piezoelectric nerve guidance conduits convert passive tubular bridges into active electrophysiological modulators. The defining advantage of biomechanical energy-harvesting systems in peripheral repair is their capacity to couple regenerative stimulation directly to patient locomotion, providing a battery-free, activity-dependent paradigm that requires no external hardware. The primary limitation, however, is the variable nature of *in vivo* mechanical inputs, which may yield inconsistent electrical dosing. While conductive–piezoelectric hybrids address charge distribution and oxidative burden simultaneously, heterotypic bilayer interfaces are vulnerable to delamination under cyclic tensile strains imposed by limb movement. Systems incorporating transient metals (Mg, Ag) must further control corrosion kinetics to prevent localized cytotoxic ion accumulation. Future designs should employ multi-material additive manufacturing to produce monolithic, gradient-structured scaffolds integrating capacitive charge-storage elements, enabling conversion of irregular mechanical inputs into consistent, physiologically appropriate neuroregenerative signals.

## Conclusions and prospects

Electroactive biomaterials, including conductive and piezoelectric materials, have fundamentally transformed neural regeneration strategies, shifting the field from passive structural scaffolding toward active bioelectric modulation [22, 24]. By replicating or autonomously generating physiological electrical cues, these platforms interface with the post-injury microenvironment to restore transmembrane ionic gradients and correct the pathological neural imbalances in both CNS and PNS injury [15, 39, 86]. Current systems demonstrate substantial preclinical efficacy, activating VGCCs and engaging downstream calcium-dependent signaling cascades to promote axonal elongation and myelination [149, 197, 243]. The apparent conflict between the destructive Ca^2+^ influx in SCI and the pro-regenerative Ca^2+^ signaling in treatment resolves once the spatiotemporal profile of the signal is specified. Pathological Ca^2+^ overload follows sustained excitotoxic stimulation and chronic VGCC upregulation, producing high-amplitude, prolonged, and spatially diffuse elevations in intracellular Ca^2+^. These elevations exceed mitochondrial buffering capacity, activate calpain, drive excess ROS production and commit affected cells to apoptotic pathways that block tissue repair. Therapeutic Ca^2+^ signaling, generated by electroactive biomaterials or localized ES, takes a distinct form: brief, low-to-moderate amplitude oscillations confined to discrete microdomains such as those within axonal growth cones. These transients activate calmodulin/CaMKII signaling and transcription factors including CREB, supporting actin cytoskeletal remodeling, directed axon extension and neuronal survival. Shifting the injured cellular microenvironment from a sustained global overload to a bounded, pulsatile signaling regime directly engages endogenous repair mechanisms. Nevertheless, significant gaps remain in our understanding of the broader ionic homeostasis governed by these materials. While most studies have focused on intracellular calcium dynamics, the post-implantation regulatory status of other critical ion channels, including functionally suppressed potassium channels [19, 123] and dysregulated chloride cotransporters such as KCC2 and NKCC1 [66, 68], remains poorly characterized. To understand how electroactive materials might influence these targets, conventional ES offers a plausible mechanistic bridge. Existing research indicates that ES directly modulates chloride homeostasis. While certain stimulation paradigms have been associated with NKCC1 upregulation [245, 246], targeted therapeutic ES drives the activity-dependent release of BDNF. This local BDNF secretion subsequently upregulates KCC2, restoring the physiological transmembrane chloride gradient and reinstating mature inhibitory GABAergic transmission. Bioelectronic interfaces actively remodel the neuroimmune microenvironment by driving microglia and macrophage polarization from a pro-inflammatory M1 state to a pro-regenerative M2 phenotype [159, 160]. Localized electrical cues from these scaffolds downregulate the secretion of neurotoxic cytokines, including TNF-α and IL-6. These signals simultaneously upregulate anti-inflammatory factors such as IL-10 and Arginase-1 [175, 157]. This phenotypic transition directly attenuates the secondary inflammatory cascade and limits the hyperactivation of reactive astrocytes in the CNS. The subsequent reduction in astrogliosis decreases the deposition of inhibitory chondroitin sulfate proteoglycans, physically and chemically softening the glial scar to permit axonal penetration [158, 159]. This cascade physically and chemically softens the glial scar to permit axonal penetration. Future work should integrate multi-omics approaches to systematically define how materials-driven electrical signals remodel the local immune microenvironment, with particular attention to microglia and macrophage polarization states, chronic neuroinflammation and glial scar formation [164, 162, 157, 220]. Electroactive biomaterials degrade while performing their electrophysiological function, making degradation kinetics and electrical output coupled design variables. Degradable scaffolds are selected by default in neural regeneration to avoid chronic compression and foreign-body reactions; this same degradation renders ion-channel engagement intrinsically transient, as hydrolytic or enzymatic cleavage progressively lowers charge-injection capacity or piezoelectric output during the repair process. Conductive oligomer-based conduits demonstrate one failure mode: percolation networks collapse abruptly once degradation crosses a threshold, deactivating VGCCs and mechanosensitive Piezo channels before axons complete bridging, and halting the Ca^2+^/MAPK cascades required for Schwann cell myelination. Transient metallic electroceuticals based on Mg or Zn show the reverse problem: rapid corrosion releases hydrogen gas and raises local pH, gating pH-sensitive channels abnormally and causing localized cytotoxicity. Both cases identify degradation rate as a primary design parameter: block copolymers and alloyed nanocoatings can be tuned so that electrical attenuation kinetics track the temporal course of axonal circuit restoration, delivering ion-channel stimuli of appropriate magnitude until endogenous bioelectric homeostasis is restored.

Despite substantial preclinical progress, clinical translation remains constrained by persistent challenges, including interface instability, uncontrolled degradation kinetics and the lack of standardized evaluation frameworks. The clinical success of passive nerve guidance conduits, including FDA-approved collagen-based conduits such as NeuraGen® for bridging short peripheral nerve gaps, establishes an important reference point for biomaterial-mediated neural repair [247]. However, experience from peripheral nerve repair suggests that passive structural support alone is insufficient for critical-size defects. This limitation becomes even more pronounced in CNS injuries, where inhibitory glial scar formation and severe electrophysiological disruption present additional barriers to regeneration. The transition from passive conduits to active bioelectronic interfaces incorporating conductive or piezoelectric scaffolds introduces additional translational challenges. Chronic implantation of electroactive constructs frequently elicits a foreign-body response, resulting in fibrotic encapsulation, progressive material degradation or dopant leaching and rising interfacial impedance that degrades long-term electrochemical performance [24, 143, 146]. Beyond material stability, electroactive implants intended for clinical use must also satisfy practical surgical requirements, including suturability and intraoperative handling, and exhibit degradation kinetics matched to the pace of human neural tissue remodeling, a constraint less demanding in rodent models given their accelerated regenerative timelines. Addressing these limitations will require moving beyond conventional small-animal models, which inadequately replicate human anatomical dimensions, neuroimmune responses and rehabilitation timescales. Establishing clinical viability will therefore depend on standardized long-term evaluation protocols in large-animal models, such as non-human primates or swine, together with clear regulatory frameworks that define acceptable charge-injection limits, systemic biosafety thresholds and chronic biocompatibility criteria for next-generation electroactive implants.

Beyond regulatory and evaluation challenges, progress will also depend on advances in material design and broader technological integration. Next-generation platforms should transition from static constructs to spatiotemporally adaptive systems, in which ES profiles and biodegradation kinetics are coordinated with the progression of tissue repair and functional circuit reestablishment. Nanoscale control over surface topography and local charge distribution may further enable microenvironmental regulation at the single-cell or synaptic level. Wireless, non-invasive modulation strategies, including acousto-electric and magnetoelectric approaches responsive to transcranial ultrasound or alternating magnetic fields [169, 155, 168], offer a viable route to stimulate deep cortical or spinal targets without percutaneous leads. These capabilities should ultimately converge into closed-loop bioelectronic systems capable of continuously monitoring pathological electrophysiological activity and delivering on-demand electrical or molecular interventions. Integration of artificial intelligence and machine learning to guide the design of biomimetic matrices with defined piezoelectric coefficients and conductivity profiles will further accelerate material optimization. Taken together, these advances hold the potential to establish electroactive biomaterials as clinically viable tools for functional neurological recovery.
